# Breadfruit flour is a healthy option for modern foods and food security

**DOI:** 10.1371/journal.pone.0236300

**Published:** 2020-07-23

**Authors:** Ying Liu, Paula N. Brown, Diane Ragone, Deanna L. Gibson, Susan J. Murch

**Affiliations:** 1 Chemistry, University of British Columbia, Kelowna, British Columbia, Canada; 2 Natural Health and Food Products Research Group, British Columbia Institute of Technology, Burnaby, British Columbia, Canada; 3 Breadfruit Institute, National Tropical Botanical Garden, Kauai, Hawaii, United States of America; 4 Biology, University of British Columbia, Kelowna, British Columbia, Canada; Embrapa Agroindústria Tropical, BRAZIL

## Abstract

Breadfruit is a traditional staple crop from Pacific islands with the potential to improve worldwide food security and mitigate diabetes. Flour produced from breadfruit is a gluten-free, low glycemic index, nutrient dense and complete protein option for modern foods but basic scientific knowledge of health impacts of a breadfruit-based diet in animals and humans was lacking. We designed a series of studies to provide basic and fundamental data on impacts of a breadfruit-based diet through an *in vitro* and *in vivo* model. Cooked breadfruit flour was digested through a multi-stage enzyme digestion model to estimate protein digestibility in comparison to wheat flour. Breadfruit protein was found to be easier to digest than wheat protein in the enzyme digestion model. The flour digestions were applied to Caco-2 cells to test the cytotoxicity and to measure the immunogenicity through cytokine expression. No significant differences were observed for immune factors and cytokines (IL-4, IL-10, IL-8, TNF-α, IFN-γ) on Caco-2 cells between the breadfruit and wheat groups. A breadfruit-based rodent chow was formulated by substitution of all of the wheat in the standard formulation with breadfruit. The diets were isocaloric, nutrient equivalent and used to feed male and female C57BL/6 mice for 21 days. No sign of malnutrition, discomfort, illness or death was observed among the mice because of the diet. The histology and the cytokine expression of the mice ileum from both groups were analyzed and showed similar results. The expression of major bacteria was measured in the colon and showed similar results. Mice fed the breadfruit diet had a significantly higher growth rate and body weight than standard diet fed mice. No negative health outcomes were observed in studies with *in vitro* or in vivo models and breadfruit flour is a healthy alternative to other starches for modern foods.

## Introduction

Breadfruit (*Artocarpus altilis*) is a traditional staple crop that most likely originated in Borneo [1] and was carried throughout the Pacific Islands with voyaging canoes [1, 2]. With high annual production (>400 kg per tree) [1, 3], and newly developed tissue culture propagation methods [4, 5], breadfruit is considered to be one of the best candidate plants to distribute to undernourished populations in tropical areas for cultivation as a staple [6, 7]. It is often used as a potato substitute in dishes as the fresh fruit can be baked, steamed, boiled, fried, microwaved, grilled, and barbecued [1, 2]. Our previous studies demonstrated that breadfruit protein contains all of the essential amino acids and is especially rich in phenylalanine, leucine, isoleucine and valine [7]. The most cultivar “Ma’afala” has been planted in nearly 50 countries in the last decade and is an excellent food resource with higher total essential amino acid content than other staples including wheat, corn, rice, potato, soybean and yellow pea [1, 7]. Research on breadfruit starch has revealed its advantages over wheat flour on water and oil holding capacity, swelling power, and viscosity [8, 9]. Moreover, cooking process was found to cause little alteration in the bioactive compounds of breadfruit and water was the best extractor compared to organic solvent, making it a promising functional ingredient and a great substitute for wheat in the processed food products [10].

Although breadfruit has been consumed by humans for thousands of years, detailed and systematic studies of the health impacts of a breadfruit diet have not previously been conducted. Human studies related to breadfruit in the diet have mainly focused on the glycemic index (GI) measurement. Several researchers [11–13], indicated that breadfruit had a low glycemic index as compared to many common staples such as wheat, cassava, yam and potatoes. However, the literature is somewhat complicated by a study in 1995 that reported the death of four male Hooded Lister rats after consumption of breadfruit seed extracts [14] that led to the classification of breadfruit as a “hazardous plant” by the United States Food and Drug Administration (FDA). The study indicated that the seeds were obtained from an *Artocarpus altilis* tree at the Mayaguez Institute of Tropical Agriculture (Puerto Rico) but the records indicated that the varieties of breadfruit at the institute were mostly seedless [15] suggesting that the species was likely misidentified. Aka et al [16] reported that a raw and partially raw breadfruit diet induced severe weight loss in male rats but cooked breadfruit resulted in weight gain. Unfortunately, the reported methods were not clear with respect to the incorporation of breadfruit either as a supplement along with the control diet or processed into the diet and the diet composition was not analyzed, making it difficult to understand exactly what the mice were consuming [16]. Adepeju et al., [17] also report weight loss in albino weanling rats fed diets with various ratios of breadfruit, soy and groundnuts but the methods of preparing the diets were not described and the nutrient composition was not reported.

The objective of the current study was to determine whether a diet containing breadfruit flour poses any serious health concerns. The specific objectives were (a) to determine the digestibility of protein in breadfruit flour, (b) to determine the impacts of digested breadfruit flour on the health and viability of Caco-2 cells; which serves as a model of human intestinal barrier and (c) to assess the overall growth and health of C57BL/6 mice fed a well-formulated and -characterized standard diet that substituted breadfruit for wheat. These studies provide the basic understanding of a breadfruit-based diet for human health.

## Materials and methods

### Sample preparation

Breadfruit (cultivar: Ma’afala, *Artocarpus altilis*) fruits were harvested from a single 4-year-old-tree grown from micro-propagation at the National Tropical Botanical Garden’s McBryde Garden on Kauai, Hawaii (21°54’29.47°N; 159°30’41.45°W). No permits were required for harvest of the samples. Fruit were harvested when mature, peeled, cored and sliced with a mandolin to 6.35 mm slices. Breadfruit slices were dried overnight at 115°C in an Excalibur Dehydrator to complete dryness and shipped to University of British Columbia Okanagan campus. Flour was prepared by grinding dried breadfruit with a burr-style coffee grinder (Bunn, Illinois, USA) controlling for excess heat. Wheat flour (all purpose, Robin Hood™) was purchased from a local supermarket.

### Multi-stage enzyme digestion model to mimic human digestion

**Fig 1:**
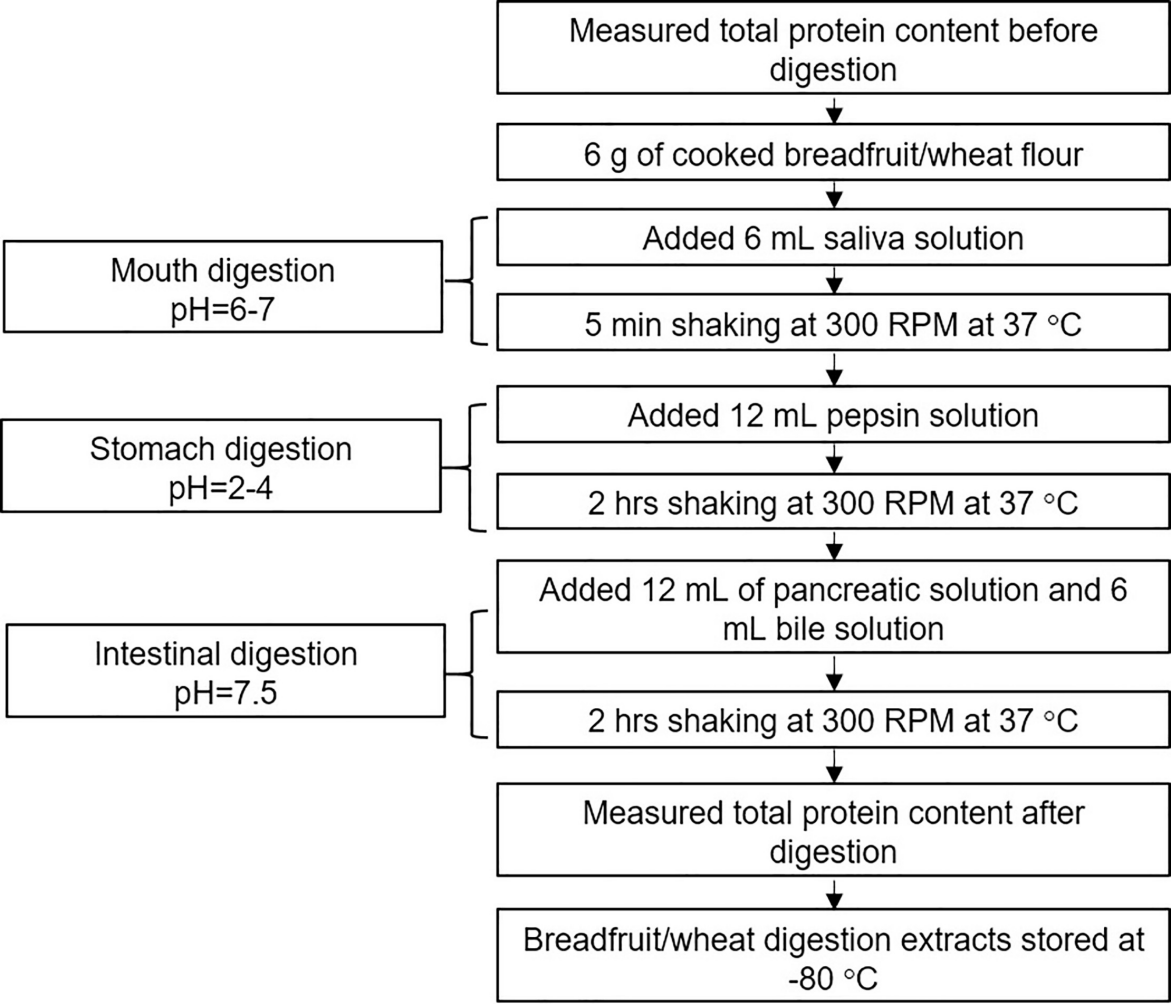
A schematic representation of the *in vitro* digestion model.

The multi-stage enzyme digestion model for digesting breadfruit and wheat was modified based on the previous methods [18–20]. The digestion process was divided by mouth digestion, stomach digestion, and intestinal digestion (Fig 1; S1 Table). Mouth digestion was mimicked by mixing 6 g of cooked (water boiled) flour with 6 mL artificial saliva. The pH of the solution was adjusted to 6–7 with HCl (1N), using a pH meter. The solution was incubated at 37°C for 5 min in an incubator (Innova^®^44, Eppendorf, Connecticut, USA). The solution was then mixed with 12 mL pepsin solution. The pH of the mixture was adjusted to 2–4 with HCl (6N). The mixture was incubated at 37°C and shaken consistently at 300 RPM for 2 hours. The intestinal digestion was performed by adjusting the solution pH to 7.5 with NaOH (1N) and then mixing the solution with 12 mL of pancreatic solution and 6 mL of bile solution. The solution was incubated at 37°C and shaken consistently at 300 RPM for 2 hours. The compositions of saliva, pepsin solution, pancreatic solution and bile solution are listed in supplementary material. The digestion samples were adjusted to pH 7.5. The digestion extracts were stored at -80°C before analysis. A set of digestion reactions was conducted following the same digestion process (Fig 1) in absence of flour samples to obtain a digestive enzyme solution that contains all the enzymes and buffers used in the process.

### Protein determination in the flours and digestion extracts

Protein was determined by two orthogonal methods to avoid incorrect determinations arising from unidentified interferences as described by Jones et al. [2]. In brief, 10 mg flour was extracted in 0.5 mL protein extraction buffer (20 mM HEPES, 150 mM NaCl, 0.3% Tween 20) with 15 min sonication at room temperature. The tubes were centrifuged for 10 min at 13,000 RPM. The supernatant was saved for the protein determination of flours. The protein content of flours and digestion extracts were first determined by BCA (bicinchoninic acid) protein assay (Thermo Scientific, Manassas, USA) and then determined by a modified Lowry Protein Assay kit (Thermo Scientific), following standard kit instructions. The protein content of digestion extracts was measured using the same protocol with a 1:5 dilution of the digestion extract in 0.9% NaCl prior to colorimetric determinations.

### Effects of breadfruit digestion on Caco-2 cells: Cell viability

For the cell culture assay, Caco-2 cells were obtained from the American Type Culture (ATCC^®^ HTB-37^TM^) and established according to previous protocol [21]. The digestion extracts were centrifuged and filtered at 13,000 RPM for 10 min before applying to Caco-2 cells. Caco-2 cells were incubated for 4 hours with four different concentrations (1%, 5%, 10%, and 50%) of three different digested extracts (digestive enzyme solution, wheat digestion solution, and breadfruit digestion solution) in the standard growth media. Cell viability was measured using a standard trypan blue method [22].

### Effects of breadfruit digestion on Caco-2 cells: Cytokine expression

Digestion extracts were applied to Caco-2 cells under 4 different conditions; non-stimulated, LPS (lipopolysaccharide) stimulated, IL-1β (Interleukin 1β) stimualted, and LPS plus IL-1β stimulated for 24 hours (Table 1). LPS from *Escherichia coli* 0111:B4 (γ-irradiated, BioXtra, suitable for cell culture; Sigma-Aldrich) was used for stimulating the cells and prepared as: 1 mg LPS was re-suspended with 1 mL nuclease-free water. 0.4 μL suspended LPS (1 mg/mL) was mixed with 9.6 μL growth medium and used for the stimulation. IL-1β (animal-component free, recombinant, expressed in *E*. *coli*, ≥98% (SDS-PAGE), ≥98% (HPLC); Sigma-Aldrich) was used for stimulating the cells and prepared as: 10 μg IL-1β was prepared by re-suspended 100 μL nuclease-free water. 0.4 μL suspended IL-1β was mixed with 9.6 μL PBS and used for the stimulation. Non-stimulated Caco-2 cells received no additional stimulations other than the digestion extracts.

**Table 1:** Experimental design for testing effects of digestion extracts combined with LPS and/or IL1β stimulation on Caco-2 cells (n = 3) after 24 hours.

Block	Stimulation	Digestion extract
**1**	Non stimulated	None
		1% Digestive enzyme solution
		1% Wheat digestion
		1% Breadfruit digestion
**2**	1000 ng/mL LPS	None
		1% Digestive enzyme solution
		1% Wheat digestion
		1% Breadfruit digestion
**3**	100 ng/mL IL 1β	None
		1% Digestive enzyme solution
		1% Wheat digestion
		1% Breadfruit digestion
**4**	1000 ng/mL LPS+ 100 ng/mL IL 1β	None
		1% Digestive enzyme solution
		1% Wheat digestion
		1% Breadfruit digestion

RNA extraction was performed on the Caco-2 cells using RiboZol, following the manufacturer’s instruction. The concentration of the RNA samples was measured by a NanoDrop 2000c UV-Vis Spectrophotometer (Thermo Scientific). 1 to 2 μg of RNA was reversed transcribed using an iScript cDNA synthesis kit based on manufacturer’s instructions An Ssofast^TM^ EvaGreen Supermix was used to prepare RT-qPCR (real time quantitative polymerase chain reaction) mixture. The reference gene was 18S rRNA. Gene expression of TNF-α (tumor necrosis factor-α), IFN-γ (interferon-γ), MCP-1 (monocyte chemoattractant protein-1), IL-10, IL-6, iNOS (inducible nitric oxide synthase), IL-8 and IL-4 were examined. All the primers were synthesized by Integrated DNA Technology (Coralville, USA) (S2 Table). Primer efficiencies were verified according to the Minimum Information for Publication of Quantitative Real-Time PCR Experiments guidelines [23]. Calculations of the expression value were performed in the Bio-Rad CFX manager 3.1 (Bio-Rad Laboratories, Ontario, Canada) using the ΔΔCt method.

### Diet design for *in vivo* mice study

A breadfruit (BF) diet was formulated based on the standard 5LG4 diet (Purina, Gary Summit, MO, USA) by replacing all the ground wheat and wheat components (~45.5% of 5LG4) with breadfruit flour (Ma’afala). Analysis of the two finished diets was conducted by a commercial lab to industry standard. The diets were isocaloric and nutritionally equivalent (S3 Table). To determine whether the breadfruit flour included non-nutrient phytochemicals that may have impacted the animal health, the antioxidant content of the diets was determined by standardized DPPH protocols [24]. The BF diet and 5LG4 diet were not significantly different in their scavenging activity at 15 minutes (S1 Fig), indicating that significant quantities of reactive phytochemicals were not present.

### Experimental animals

The animal experiments were performed by the staff at Jackson Laboratories, California, USA according to standard operating protocols. The animal protocol was approved by the University of California San Diego Institutional Animal Care and Use Committee (IACUC) to ensure the highest quality animal use and care. Mice (C57BL/6, Jackson Labs) were 7-weeks-old at the beginning of the experiment and were housed for 3 weeks in positively ventilated polycarbonate cages with HEPA-filtered air at a density of four mice per cage. The mice were ear-notched for identification. The light source was provided by using artificial fluorescent lighting, creating a 12 hour light/dark cycle. The room temperature was controlled at 22±4°C with 50±15% humidity. A total of 32 mixed sex (half male and half female) mice were randomly assigned into two groups. Each group had an equal number of female (8) and male (8) mice. Mice were housed in individually and positively ventilated polycarbonate cages with HEPA filtered air at a density of 4 mice per cage. The animal room was lighted entirely with artificial fluorescent lighting, with a controlled 12 h light/dark cycle (6 am to 6 pm light). The normal temperature and relative humidity ranges in the animal rooms were 22 ± 4°C and 50 ± 15%, respectively. The animal rooms were set to have 15 air exchanges per hour. Filtered tap water, acidified to a pH of 2.5 to 3.0 and standard rodent chow (5LG4) or experimental BF diet were provided ad libitum. Problems with the breadfruit diet crumbling in the cages meant that it was not possible to accurately determine food intake or absolute amount of food consumed.

### Overall growth evaluation and hematology analysis

Body weight, and food and water intake were measured daily at the same time. Clinical observations were made daily, examining the general health condition and to see if any skin lesions developed. At the terminal point, the mice were sacrificed by CO_2_ asphyxiation at the end of the third week using approved humane practice. Whole-body composition measurements of fat, lean, free water, and total water mass was conducted using EchoMRI-100 Analyzer (EchoMRI^TM^, USA) in the Jackson Laboratory.

### Tissue collection

The organs were weighed individually and sent as snap-frozen tissue to the UBC Okanagan. Feces were collected on days 3, 7, 14, and 21 from each cage, frozen in liquid nitrogen and then sent to the UBC Okanagan campus. One-third of the ileum sections of the mice were stored in RNAlater^®^, an RNA stabilization reagent (Qiagen^®^), and shipped frozen to the UBC Okanagan campus. A 3- to 5-mm section of the ileum was fixed in 10% buffered neutral formalin with paraffin, sectioned following standard histological methods and then stained by H&E method. The frozen tissues, feces and RNA later ileum section were stored in -80° before analysis.

### Ileum morphology examination

The morphological analysis of the H&E stained ileum slides was conducted, following the method published by Generoso et al. [25]. Villi pictures (10 pictures × 4 mice) were taken from each group using a camera (QImaging, Surrey, BC, Canada) attached to a microscope (Olympus America Inc., PA, USA) and each parameter was measured blindly using ImageJ 1.47v software (Wayne Rasband, National Institutes of Health, Maryland, USA). The following parameters were measured: distance between villi; villus height and thickness; the crypt depth; the thickness of lamina propria and epithelium: the length of mucosa, submucosa, and muscularis externa; and the number of goblet cells and red blood cells.

### Major cytokine response on ileum

RNA extraction was performed on ileum tissue of the mice, using an RNeasy^®^ fibrous tissue mini kit (Qiagen, Germany). The gene expression of TNF-α, IFN-γ, IL-10, IL-6, and iNOS was examined using RT-PCR technology, using 18S rRNA as a reference gene. The design and testing of the primers used in the study were conducted by Ghosh et al. [26].

### Quantification of bacterial groups in the colon

DNA was extracted from the frozen colon tissue of the mice using a QIAamp DNA stool mini kit (Qiagen). The production of Firmicutes members, Bacteroidetes members, Enterobacteriaceae family, *Lactobacillus* spp., and *Bifidobacterium* spp. in the colon was quantified by RT-qPCR, using Eubacteria production as a reference in the analysis. The sequence specificity of the primers was verified by Baker et al. [21].

### Feces protein, total mineral analysis, and fecal occult blood detection

Feces from the same cage and the same day were homogenized and mixed using a Coors^TM^ porcelain mortar and pestle (Sigma, USA). The protein was extracted in the protein extraction buffer before BCA analysis. The mineral content of the feces was calculated by the difference of weight of the feces before and after incineration at 600°C for 2 h in an Isotemp programmable muffle furnace (Fisher Scientific, Ottawa, ON), according to AACC 08-03.The fecal occult blood was detected using a Hemoccult Sensa^®^ kit (Beckman Coulter, USA) following the manufacturer’s protocol.

### Statistical analysis

All statistical analysis was conducted using JMP^®^ 10.0.0 (SAS Institute, Cary, NC) and GraphPad Prism 5.0 software (GraphPad Software Inc., La Jolla, CA). A series of independent two sample t-tests or two-way ANOVA analyses were conducted to determine if there were significant differences between treatments with a type 1 error rate of 0.05. Graphs were created using GraphPad Prism 5.0 software (Microsoft Corporation, Santa Rosa, CA).

## Results

### Protein digestibility

**Fig 2:**
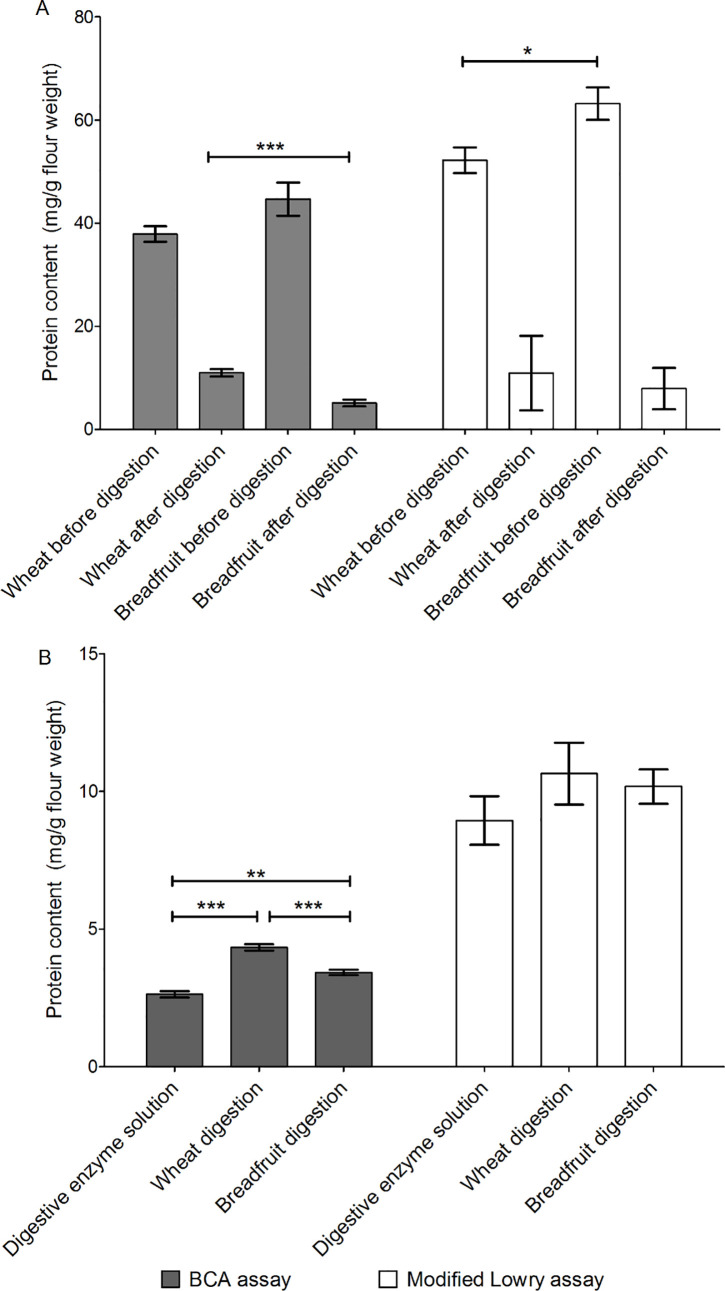
Protein content measured by BCA and modified Lowry assays. (A) Wheat and breadfruit flour before and after digestion. (B) Digestion extracts. N = 3. * represents significant difference at α = 0.05, ** represents significant difference at α = 0.01, *** represents significant difference at α = 0.001, using two sample t-test or one-way ANOVA with Tukey-Kramer Honest Significant Difference (HSD) test. Bar represents the standard error of 6 replicates within each treatment.

To eliminate the possibility of inaccuracies in the measurement of protein content in the flour and digestions, two orthogonal protein measures were made at each step. The absolute values of protein were different between the two methods, but the overall trends were consistent. Overall, about 87% (modified Lowry assay) or 89% (BCA assay) of the breadfruit protein was fully digested in the *in vitro* digestion model while 79% (modified Lowry assay) or 71% (BCA assay) of the wheat protein was fully digested (Fig 2A). After the digestion reactions, there was significantly more intact protein in the wheat digestion extract than the breadfruit confirming that more of the breadfruit protein could be digested (Fig 2A). Both breadfruit and wheat digestions had significantly higher protein than the digestive juice based on BCA assay; however, this difference was not significant using modified Lowry assay (Fig 2B).

### *In vitro* evaluation of breadfruit cytotoxicity through cell viability

**Fig 3:**
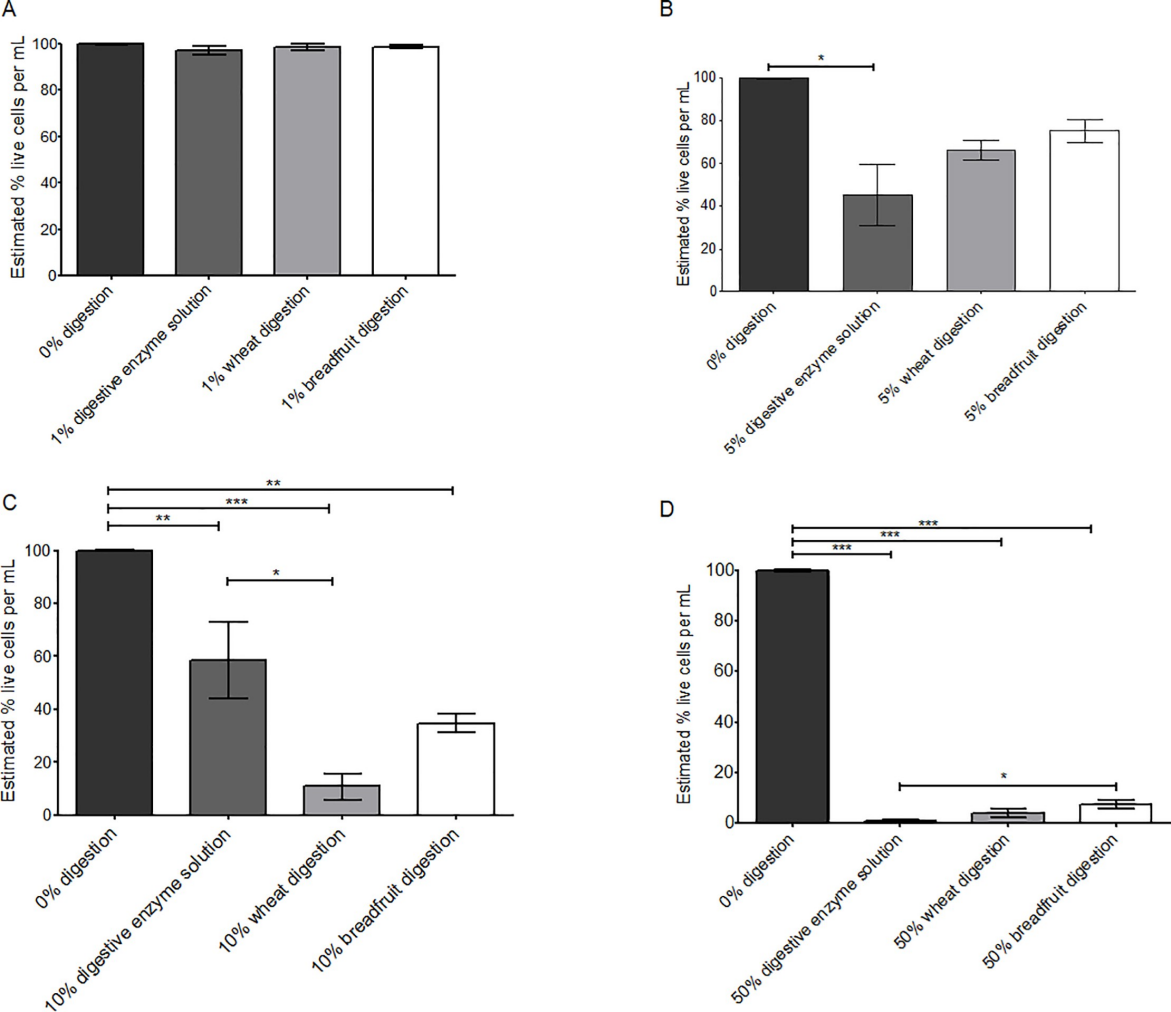
Effects of digestion extracts of wheat and breadfruit on cell viability. (A) 1% digestion extracts. (B) 5% digestion extracts. (C) 10% digestion extract. (D) 50% digestion extracts. Error bars represent the standard error of 3 replicates within each treatment. * represents significant difference at α = 0.05, ** represents significant difference at α = 0.01, *** represents significant difference at α = 0.001, using one-way ANOVA with Tukey-Kramer Honest Significant Difference (HSD) test.

There was no significant difference in the viability of untreated cells and cells treated for 4 hours with flour digestion extracts (1% of either digestive enzyme solution, wheat digestion or breadfruit digestion) (Fig 3A). When the digestion extracts were included at 5% in the cell cultures, breadfruit digestion treated cells had significantly increased viability relative to both the no digestion and wheat digestion treated cells (Fig 3B). When the digestion extract was included at 10% in the cell culture medium, cell viability was significantly reduced in all treatments (Fig 3C). At 50% of digestion extracts in the cell cultures, the breadfruit digestion extract had a significantly higher cell viability (42% higher) than the digestive enzyme solution without flours (Fig 3D). Overall, Caco-2 cells exposed to breadfruit digestion extracts had a significantly higher cell viability than cells exposed to the digestive enzyme solution alone. Increasing the amount of any of the dilution extracts significantly reduced the absolute number of viable cells and the 1% digestion extracts was most suitable for comparative studies.

### *In vitro* evaluation of breadfruit immunogenicity through cytokine expression

**Fig 4:**
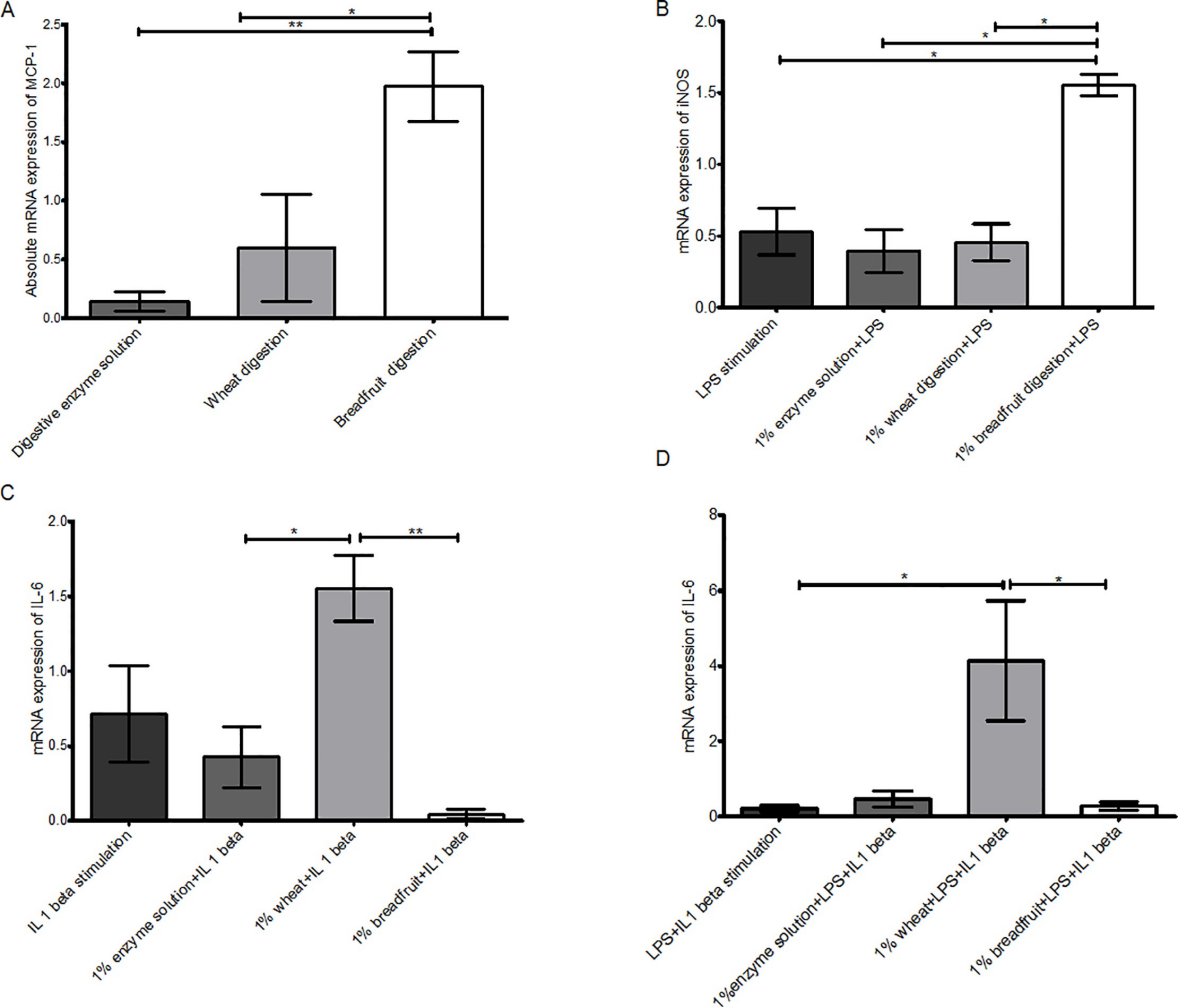
Cytokine responses of Caco-2 cells towards digestion treatments under different stimulations. (A) non stimulated. (B) LPS stimulated (C) IL-1 beta stimulated (D) LPS plus IL 1 beta. Error bars represent the standard error of 3 replicates within each treatment. * represents significant difference at α = 0.05, ** represents significant difference at α = 0.01, *** represents significant difference at α = 0.001, using one-way ANOVA with Tukey-Kramer Honest Significant Difference (HSD) test.

Breadfruit flour digestion triggered a cytokine response on Caco-2 cells similar to wheat flour digestion. All eight cytokines expression were examined, but no significant differences were observed for IL-4, IL-10, IL-8, TNF-α or IFN-γ between wheat digestion treated group and breadfruit digestion treated group (S2–S5 Figs). For digestion extract treated unstimulated cells, 1% breadfruit treated cells had significantly higher MCP-1 expression than wheat-treated cells and digestive enzyme solution treated cells (Fig 4A). When LPS stimulation was applied with 1% digestion extracts, cells exposed to the breadfruit digestive extract had a higher iNOS expression than other groups (Fig 4B). When IL-1β stimulation was applied, the 1% wheat digestion extract stimulated a significant higher IL-6 response than the breadfruit group (Fig 4C). When the LPS and IL-1β stimulations were combined, the expression of IL-6 was significantly higher in wheat treated cells than breadfruit treated cells (Fig 4D).

### Impact of breadfruit diet on mice overall health and growth

**Fig 5:**
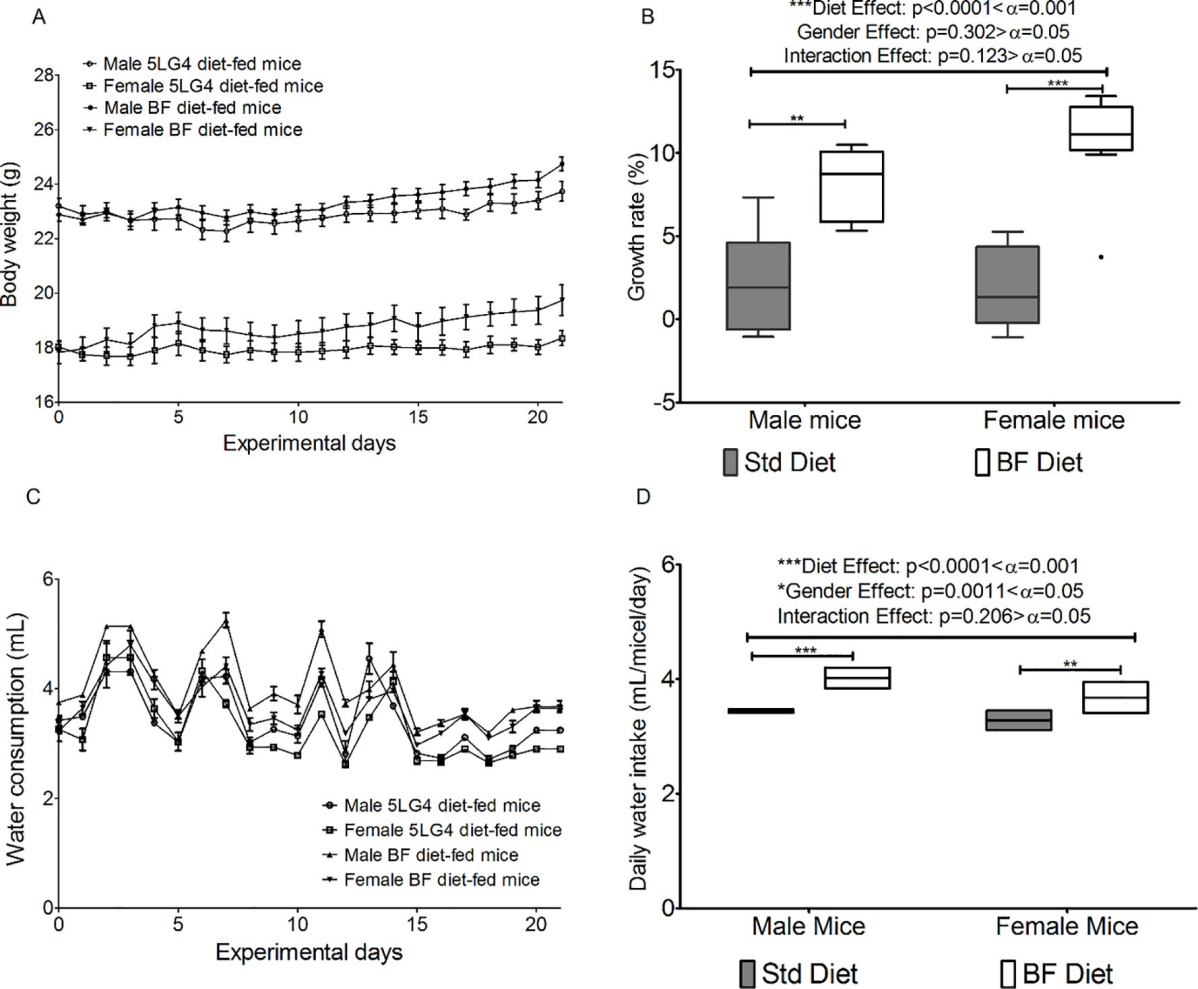
Comparison of growth parameters on mice fed on breadfruit (BF) and 5LG4 diets. (A) Daily body weight. (B) Growth rate. (C) Daily water consumption. (D) Average daily water consumption. Bars represents the standard error of the measurement among eight mice from the same group. * represents significant difference at α = 0.05, ** represents significant difference at α = 0.01, *** represents significant difference at α = 0.001.

During the three-week-trial, there was no report of death or any abnormal clinical symptoms for both male and female BF-fed (breadfruit-fed) mice. Both BF- and 5LG4-fed mice showed similar growth patterns (Fig 5A). Three weeks of the breadfruit diet resulted in a slightly higher growth rate (7.32% overall, 5.82% male, 8.82% female) in mice than the 5LG4 diet (Fig 5B), with an equal effect on both male and female mice. Since the breadfruit diet was very crumbly, the food loss during the trial was significant, and more breadfruit diet was used in the feeding, therefore the usage cannot be interpreted as the food intake. There was a significant higher daily water consumption (12.7% overall, 14.4% male, 10.6% female) in mice fed on the breadfruit diet compared to mice fed on the 5LG4 diet (Fig 5C & 5D), but there was no interaction between diet and gender. Both the growth rate and daily water intake were very significantly impacted by diets.

At the end of the three-week-trial, the body composition was similar between BF- and 5LG4-diet-fed mice (Table 2; S4 Table). Male mice consuming the BF diet had significantly higher body weight (4% higher) and lower fat (2.2% less) than male mice consuming the 5LG4 diet (Table 2). There were few significant differences in the weights of various tissues and organs in response to the diet. Female BF-fed mice had significantly larger duodena and male BF-fed mice had significantly heavier femurs (S4 Table), while all the other organs, including the brain, heart, liver, kidney, and skin were not significantly different in weight (Table 2).

**Table 2:** Differences in body composition and tissue weight between the breadfruit-fed (BF-fed) mice and 5LG4-fed mice.

Sex	5LG4 diet	BF diet	Male mice		Female mice			p	
Diet			5LG4 diet	BF diet	5LG4 Diet	BF Diet	Diet	Gender	Diet*Gender
Body weight (g)	21.0±0.7	22.2±0.7	**23.7±0.4**^**a**^	**24.7±0.3**^**b**^	18.4±0.3	19.7±0.6	0.0042	<0.0001	0.629
Fat	17.1%±0.7%	15.7%±1.0%	**15.0%±0.5%**^**a**^	**12.8%±0.6%**^**b**^	19.1%±0.7%	18.5%±1.2%	0.0924	<0.0001	0.321
Lean	73.4%±0.9%	72.9%±0.9%	75.4%±1.1%	74.7%±0.7%	71.5%±0.9%	71.1%±1.5%	0.631	0.0016	0.908
Free water	0.5%±0.1%	0.5%±0.1%	0.5%±0.1%	0.4%±0.0%	0.4%±0.1%	0.6%±0.1%	0.741	0.664	0.0692
Total water	60.7%±0.7%	60.4%±0.8%	62.3%±0.9%	62.0%±0.5%	59.0%±0.8%	58.9%±1.3%	0.817	0.0013	0.926
Brain (g)	0.45±0.004	0.44±0.007	0.46±0.003	0.45±0.006	0.44±0.006	0.43±0.013	0.195	0.0461	0.661
Heart (g)	0.12±0.003	0.12±0.005	0.13±0.004	0.13±0.006	0.11±0.004	0.11±0.005	0.580	0.0002	0.662
Kidney (g)	0.25±0.009	0.26±0.008	0.28±0.009	0.29±0.006	0.22±0.008	0.24±0.008	0.232	<0.0001	0.736
Lungs (g)	0.23±0.010	0.22±0.010	0.24±0.011	0.22±0.010	0.21±0.014	0.21±0.018	0.397	0.003	0.0795
Liver (g)	0.94±0.052	0.88±0.066	1.12±0.033	0.93±0.126	0.76±0.033	0.83±0.047	0.505	0.194	0.589
Skin (g)	0.009±0.003	0.007±0.001	0.013±0.006	0.008±0.001	0.005±0.000	0.006±0.001	0.576	0.0134	0.371

Values highlighted in bold and with a superscript letter in each section are significant difference at alpha = 0.05, using 2 sample t test. p value was calculated using two-way ANOVA analysis

### Ileum morphology examination

**Fig 6:**
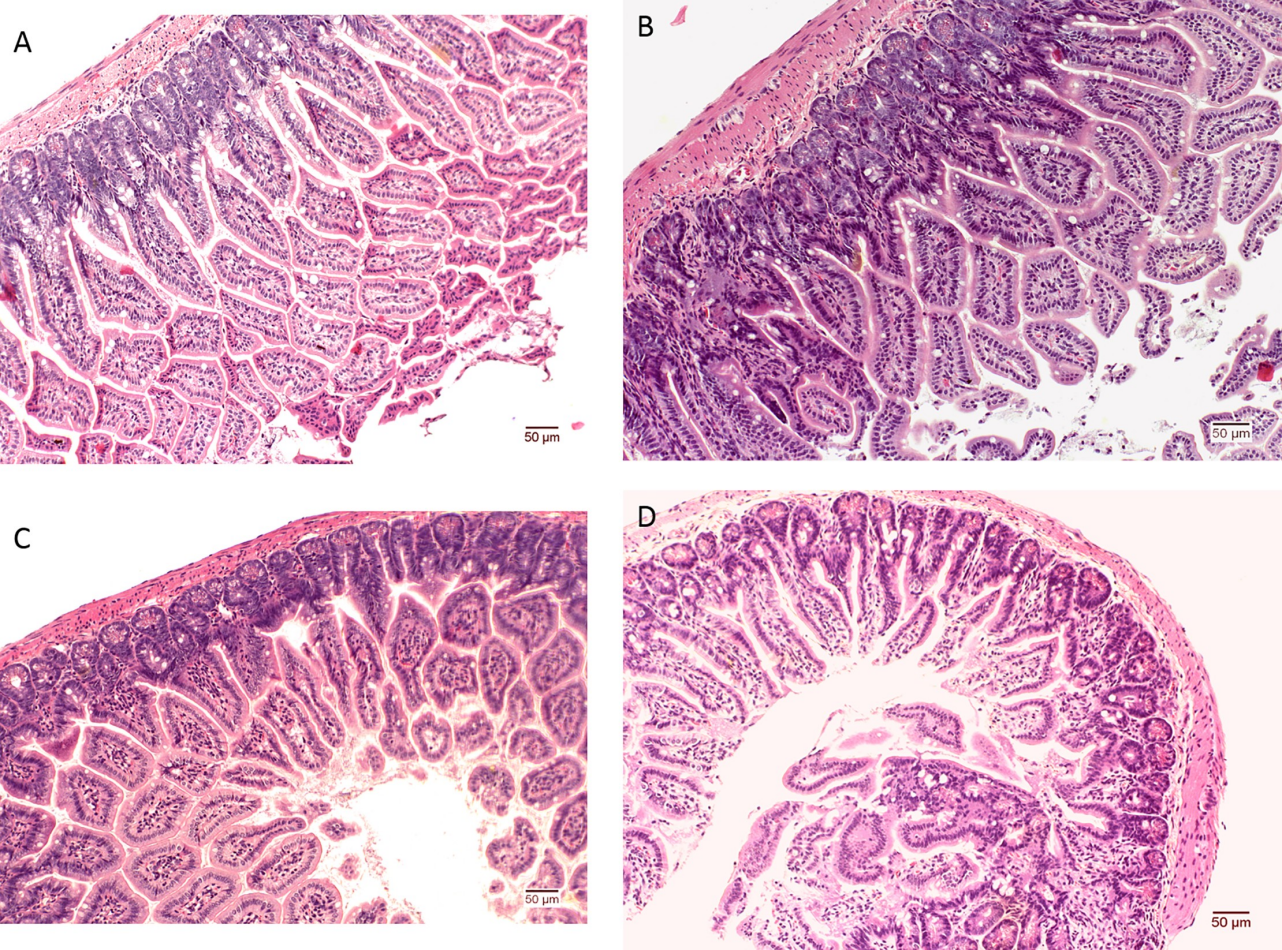
Representative histopathology of the ileum tissues stained with haematoxylin and eosin (H&E). (A) 5LG4-fed male mice. (B) BF-fed male mice. (C) 5LG4-fed female mice. (D) BF-fed female mice. Scale bar is 50 μm.

Mice from both diet groups had no significant difference in the villus height and thickness; distance between the villi; crypt depth; thickness of lamina propria and epithelium; length of mucosa, submucosa, and muscularis externa; and number of goblet cells and red blood cells (S5 Table). There was no difference in the ileum morphology between 5LG4-fed and BF-fed mice (Fig 6).

### Major cytokine responses on ileum

**Fig 7:**
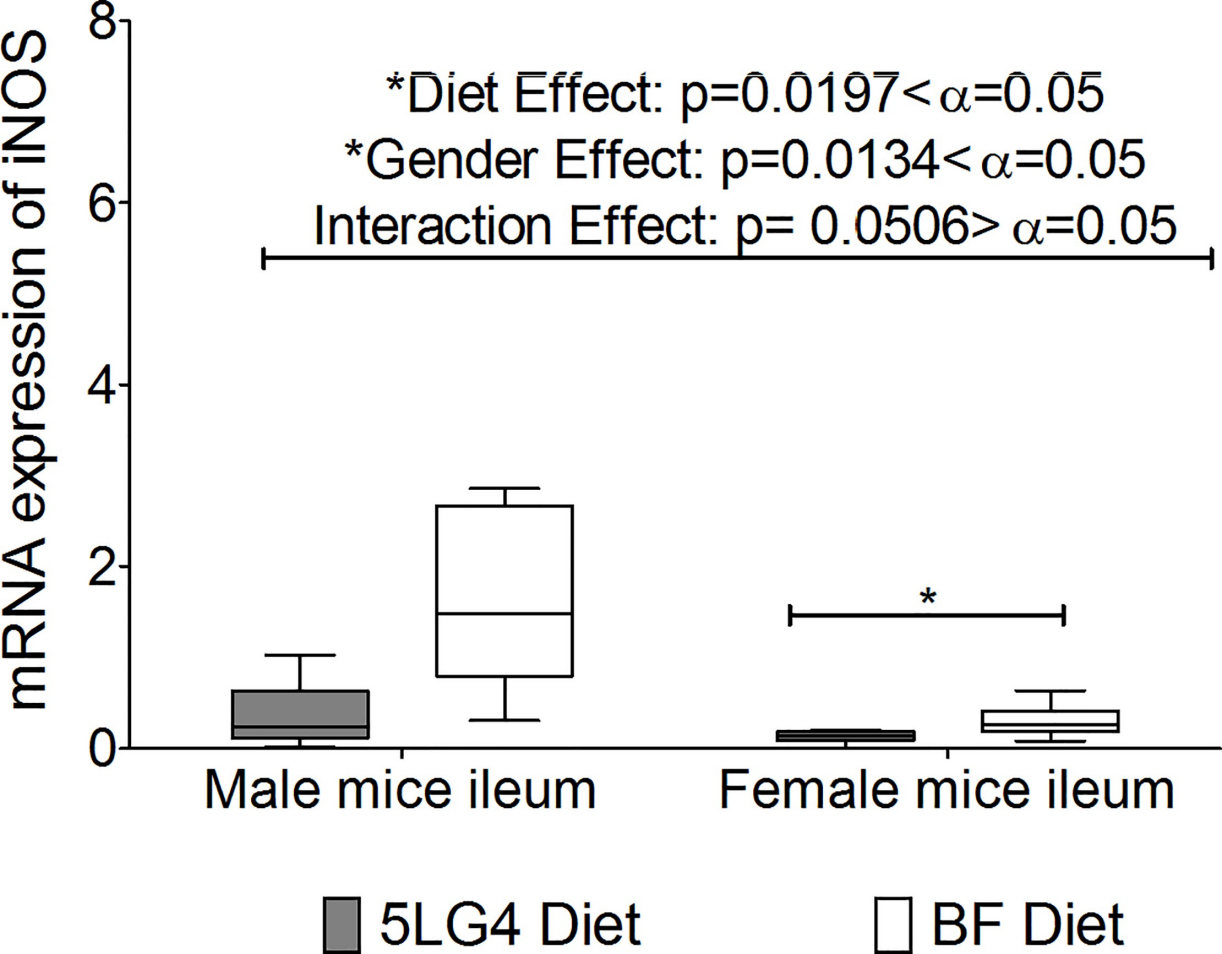
Comparison of iNOS expression between ileums of breadfruit (BF)-and 5LG4-fed mice. * represents a significant difference between the two groups using 2 sample t-test at alpha level 0.05. Middle line denotes media, whiskers are determined by Tukey method, which represent largest value below 75^th^ percentile plus 1.5 times interquartile distance IQR or lowest value above 25^th^ percentile minus 1.5 times IQR.

Cytokines expression, including TNF-α, IL-10, IL 6, and IFN-γ, were examined in the ileum tissue of the mice and no significant difference was found as a result of consumption of the 5LG4 or breadfruit diet (S6 Fig). Diets had a significant impact on iNOS expression (Fig 7). Female BF-fed mice had significantly higher iNOS expression than female 5LG4-fed mice (Fig 7); however, the difference was less than 0.5-fold. BF-fed mice had very similar cytokine responses to 5LG4-fed mice in their ileums.

**Fig 8:**
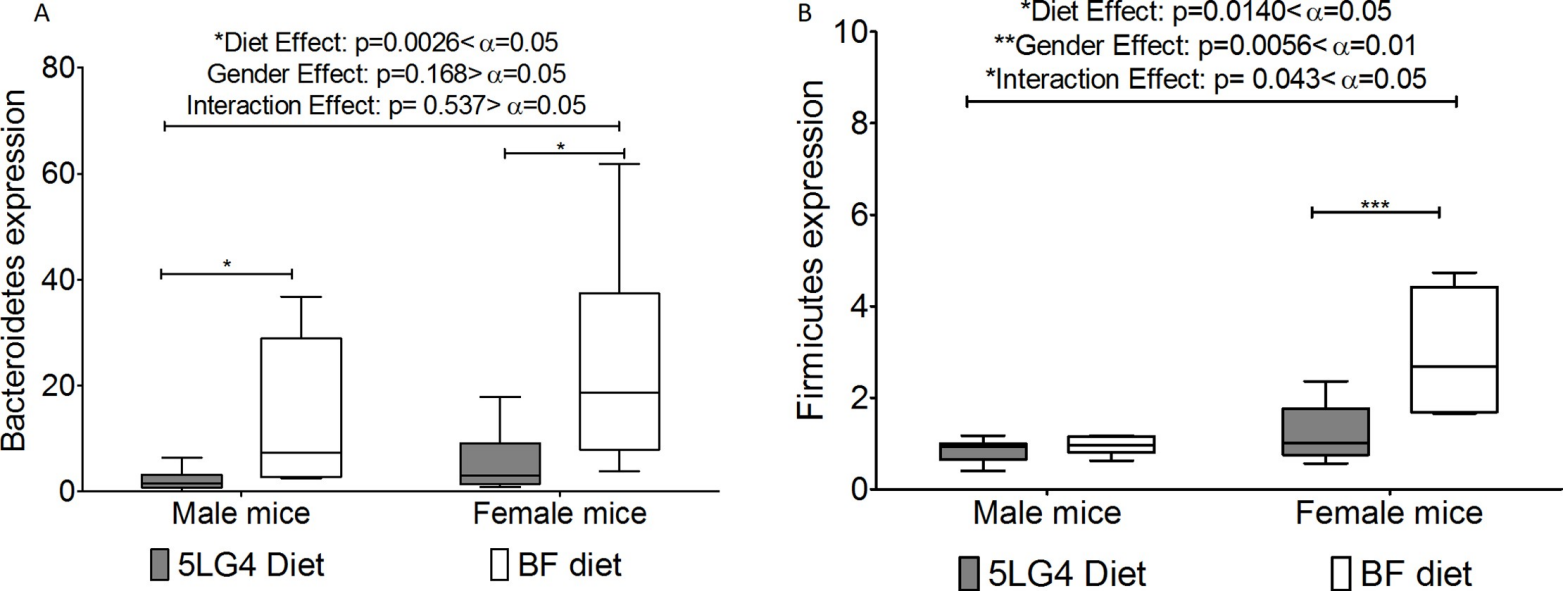
The comparison of bacteria expression between ileums of breadfruit diet and 5LG4 diet fed mice. (A) Bacterioidetes. (B) Firmicutes. *, **, *** represents significant difference between the two groups using 2 sample t-test at alpha level 0.05, 0.01, 0.001, respectively. Middle line denotes media, whiskers are determined by Tukey method, which represent largest value below 75^th^ percentile plus 1.5 times interquartile distance (IQR) or lowest value above 25^th^ percentile minus 1.5 times IQR.

Gene expression of bacterial groups, *Bifidobacterium* spp., *Lactobacillus* spp., and Enterobacteriaceae in the colon were compared and no significant differences were found between 5LG4-fed and BF-fed mice (S7 Fig). Both male and female mice fed on the breadfruit diet showed a higher gene expression of Bacteroidetes than mice fed on the 5LG4 diet (Fig 8A). Female BF-fed mice showed a higher gene expression of Firmicutes (Fig 8B). Diets had a significant impact on the gene expression of Bacteroidetes and Firmicutes. However, the ratio between Firmicutes and Bacteroidetes was not significantly different between the two diets (male 5LG4-fed mice: 0.61±0.17, male BF-fed mice: 0.20±0.05, female 5LG4-fed mice: 0.45±0.12, female BF-fed mice: 0.30±0.13) (Fig 8). Other bacteria groups studied were not significant between the diet groups.

### Fecal protein, total mineral analysis, and fecal blood detection

**Fig 9:**
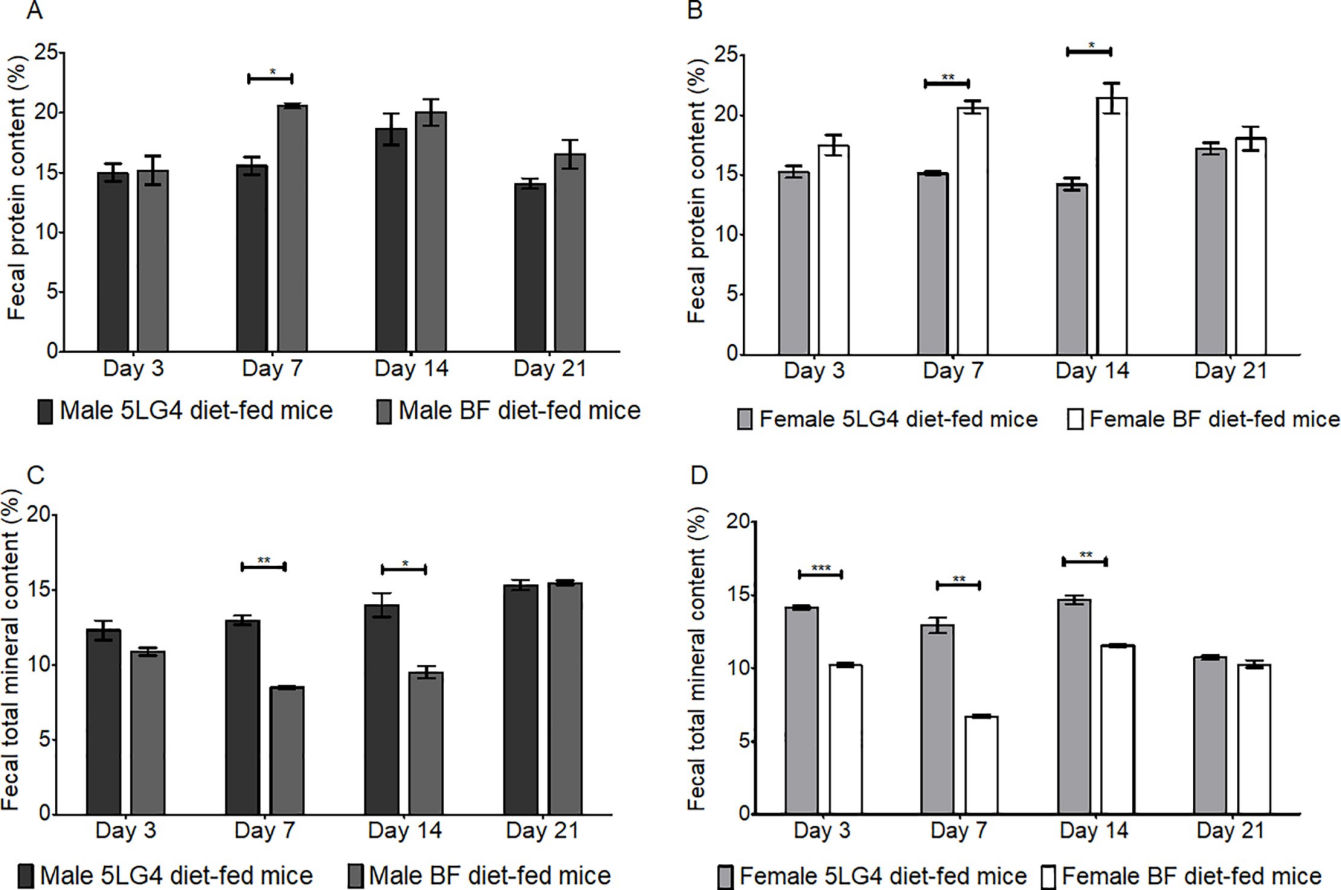
Comparison of fecal protein and total mineral content between breadfruit and 5LG4 diet fed mice. (A) Fecal protein content in male mice. (B) Fecal protein content in female mice. (C) Fecal total mineral content in male mice. (D) Fecal total mineral content in female mice. Measurements were taken at Day 4, Day 7, Day 14, and Day 21. *, **, *** represent a significant difference between the two groups using 2 sample t-test at alpha level 0.05, 0.01, 0.001, respectively. Bars represent the standard error of the measurement among eight mice from the same group.

Feces collected from the BF-fed mice tended to have a higher protein content than the 5LG4-fed mice (Fig 9A & 9B). Both male and female BF-fed mice showed a significantly higher fecal protein than 5LG4-fed mice at Day 7 (Fig 9A & 9B). In terms of total mineral content, however, the breadfruit diet fed mice showed a trend of lower percentage (1–6%) than the 5LG4 diet fed mice, especially at Day 7 and 14, where both male and female BF-fed mice had significantly lower fecal mineral content than 5LG4 fed mice (Fig 9C & 9D). By the end of the three-week trial (Day 21), there was no significant difference in the fecal protein or mineral content between the two diet groups. Regardless of the collection date, gender, and diet, the fecal blood detection showed that there was no blood in all of the feces collected.

## Discussion

The motivation of this project was to contribute to the development of breadfruit as a sustainable, environmentally friendly, and high-production crop in developing countries and tropical areas to feed and support peoples indigenous to these areas. Fundamental understanding of the health impact of breadfruit digestion and breadfruit diets is necessary and imperative to establishment of breadfruit as staple or as a functional food in the future.

As the first complete, fully-designed breadfruit diet study, our data showed that breadfruit diet does not impose any toxic impact on Caco-2 cells or mice. We did not identify any factors that might indicate potential for allergic or adverse reactions in either humans or animals. There was no reported death, sickness, or malnutrition of the mice after consuming a breadfruit diet for 3 weeks. Similar to our findings, human studies related to breadfruit have not reported any discomfort or death after consuming breadfruit [11–13, 27]. This is also supported by the long history of breadfruit consumption by Pacific Island populations [1, 28]. Unfortunately, the breadfruit chow crumbled making it difficult to estimate the loss during feeding and food intake. It is possible the mice consumed less/equal amount of breadfruit diet but end up with better growth compared to wheat diet feed mice. Also possible those breadfruit diet mice grew better simply because they ate more. Reformulation of the chow to avoid crumbling would require additional ingredients that may also have an effect. Therefore, a different approach is needed to determine whether breadfruit in the diet increases mouse growth.

Breadfruit digestion induced a higher (~1 fold) iNOS gene expression on Caco-2 cells than the wheat digestion under LPS stimulation. This finding was observed in the *in vivo* study as well. There was a slightly higher expression of iNOS (<0.5 fold) in the ileum of mice fed on the breadfruit diet. LPS is an endotoxin found in Gram-negative bacteria, which are found to be resident in the intestinal tracts of both humans and rodents [29]. iNOS is a pro-inflammatory cytokine that can produce nitric oxide, which acts as a cytotoxic agent in the pathological process, and can regulate mucosal barrier function [30, 31]. The production of iNOS in normal mice ileum mucosa has been well demonstrated by Hoffman et al. [31], in which they explained that normal mice ileum mucosa express iNOS due to the numbers of bacteria present and this expression can vary depending on the bacterial state in the ileum. In this study, only the gene expression of the cytokines was investigated using qPCR and the absolute amounts of cytokines were not determined. In some cases, the RNA values do not accurately reflect protein levels [32] but the method is generally considered effective for determination of the relative differences between samples and treatments [32]

In a preliminary investigation of the microbiome, we examined the major bacterial groups in the colon, which are closely correlated with the bacterial groups in the ileum. The composition of the gut microbiota has been previously found to be influenced by diets [33, 34]; therefore, it is not surprising that the gut microbe populations in the phyla Bacteroidetes and Firmicutes were significantly higher in mice fed the breadfruit diet. This finding further supports the hypothesis that the iNOS expression difference in the breadfruit diet mice and control mice is a natural consequence of different bacterial populations in the ileum mucosa but further research is required to understand the full impacts of breadfruit consumption on the microbiome.

Overall, these studies support the use of breadfruit as part of a healthy, nutritionally balanced diet. The average consumption of grain in the US is 189 g (6.67 ounce) per day [35]. Consuming 189 g cooked breadfruit can meet up to near 57% of fiber, over 34% protein, vitamin C and copper, about 28% potassium and manganese, and 5.75–11.5% of iron, calcium and phosphorus of the daily recommended dietary allowances (RDA) [6, 7].

## Supporting information

S1 Table Composition of saliva, gastric solution, duodenal solution, and bile solution used in the *in vitro* digestion model to mimic human digestion. (DOCX)

S2 Table RNA sequence, best annealing temperature and primer efficiency for the primers used in the breadfruit *in vitro* cell model study. (DOCX)

S3 Table Comparison of nutritional value between the breadfruit (BF) diet and 5LG4 diet. (DOCX)

S4 Table Comparison of average body composition and tissue weight between the BF-fed (breadfruit-fed) mice and 5LG4-fed mice. (DOCX)

S5 Table Histological examination of ileum morphology. (DOCX)

S1 Fig Comparison of scavenging activity percentage at 15 minutes between breadfruit diet and 5LG4 diet using DPPH methods. Bars represents standard error calculated from the three replicates.
(DOCX)

S2 Fig Effect of digestive enzyme solution, wheat digestion and breadfruit digestion on cytokine mRNA expression in Caco-2 cells without further stimulation for 24 hours. (A) IL-10. (B). IL 8. (C) TNF-α. (D) IFN-γ. (E). IL-4. (F) iNOS. (G) IL-6.
(DOCX)

S3 Fig Effect of digestive enzyme solution, wheat digestion and breadfruit digestion on cytokine mRNA expression in Caco-2 cells with LPS stimulation for 24 hours. (A) IL-10. (B). IL 8. (C) TNF-α. (D) IFN-γ. (E). IL-4. (F) MCP-1. (G) IL-6.
(DOCX)

S4 Fig Effect of digestive enzyme solution, wheat digestion and breadfruit digestion on cytokine mRNA expression in Caco-2 cells with IL-1β stimulation for 24 hours. (A) IL-10. (B). IL 8. (C) TNF-α. (D) IFN-γ. (E). IL-4. (F) MCP-1. (G) iNOS.
(DOCX)

S5 Fig Effect of digestive enzyme solution, wheat digestion and breadfruit digestion on cytokine mRNA expression in Caco-2 cells with LPS and IL-1β stimulation for 24 hours. (A) IL-10. (B). IL 8. (C) TNF-α. (D) IFN-γ. (E). IL-4. (F) iNOS. (G) MCP-1.
(DOCX)

S6 Fig Comparison of cytokine expression between ileums of breadfruit (BF)-and 5LG4-fed mice. (A) TNF-α. (B) IL-6. (C) IFN-γ. (D) IL-10.
(DOCX)

S7 Fig The comparison of bacteria expression between ileums of breadfruit diet and 5LG4 diet fed mice. (A) Enterobacteriacae. (B) *Lactobacillus* spp. (C) *Bifidobacterium* spp.
(DOCX)
