# Supplementary material for: Breadfruit flour is a healthy option for modern foods and food security
Source: PLoS One. 2020 Jul 23;15(7):e0236300. doi: 10.1371/journal.pone.0236300 (PMC7377419; doi:10.1371/journal.pone.0236300)
Supplement: S1 Table — (DOCX) [file pone.0236300.s001.docx]

**S 1Table. Composition of saliva, gastric solution, duodenal solution, and bile solution used in the *in vitro* digestion model to mimic human digestion**

| **Saliva** | | **Gastric solution** | | **Duodenal solution** | | **Bile solution** | |
| --- | --- | --- | --- | --- | --- | --- | --- |
| Item | Concentration (g/l) | Item | g/l | Item | g/l | Item | g/l |
| KCl | 1.792 | NaCl | 5.500 | NaCl | 0.168 | NaCl | 10.518 |
| KSCN | 0.400 | NaH_2_PO_4_ | 0.533 | NaHCO_3_ | 0.081 | NaHCO_3_ | 11.570 |
| NaH_2_PO_4_ | 1.776 | KCl | 1.649 | KH_2_PO_4_ | 0.002 | KCl | 0.753 |
| Na_2_SO_4_ | 1.140 | CaCl_2_·2H_2_O | 0.799 | KCl | 0.014 | Urea | 0.500 |
| NaCl | 0.600 | NH_4_Cl | 0.612 | MgCl_2_ | 0.001 | CaCl_2_·2H_2_O | 0.444 |
| NaHCO_3_ | 3.388 | Urea | 0.170 | Urea | 0.200 | BSA | 3.600 |
| Urea | 0.400 | BSA | 2.000 | CaCl_2_·2H_2_O | 0.400 | Bile | 60.000 |
| α-amylase | 0.002 | Pepsin | 5.000 | BSA | 2.000 |  |  |
|  |  |  |  | Pancreatin | 18.000 |  |  |

urea (BioReagent Grade for molecular biology, suitable for cell culture; Sigma-Aldrich, St. Louis, MO), α-amylase from human saliva (Type XIII-A, lyophilized powder, 300-1500 units/mg protein, 1 KU; Sigma-Aldrich), pancreatin from porcine pancreas (4 x USP; Sigma-Aldrich), bile bovine (dried, unfractionated; Sigma-Aldrich), BSA (bovine serum albumin) (heat shock fraction, protease free, fatty acid free, essentially globulin free, pH=7, ≥98%; Sigma-Aldrich), pepsin from porcine gastric mucosa (lyophilized powder, 3200-4500 units/mg protein; Sigma-Aldrich)
