# Supplementary material for: Breadfruit flour is a healthy option for modern foods and food security
Source: PLoS One. 2020 Jul 23;15(7):e0236300. doi: 10.1371/journal.pone.0236300 (PMC7377419; doi:10.1371/journal.pone.0236300)
Supplement: S2 Table — (DOCX) [file pone.0236300.s002.docx]

**S 2 Table. RNA sequence, best annealing temperature and primer efficiency for the primers used in the breadfruit *in vitro* cell model study.**

| **Primer** | **Forward sequence** | **Reverse sequence** | **Best annealing temp.** | **Efficiency** |
| --- | --- | --- | --- | --- |
| 18S | GTAACCCGTTGAACCCCATT | CCATCCAATCGGTAGTAGCG | 58 °C | 100% |
| TNF-α | TCTCGAACCCCGAGTGACAA | TATCTCTCAGCTCCACGCCA | 58°C | 100% |
| IL-10 | TTCCATTCCAAGCCTGACC | CCAAGCCCAGAGACAAGATAAA | 58°C | 91% |
| IL-6 | GAGAGTAGTGAGGAACAAGCC | GGTCAGGGGTGGTTATTGC | 58°C | 92% |
| IL-4 | TTCCCCCTCTGTTCTTCCT | GTCTGTTACGGTCAACTCGG | 58°C | 95% |
| iNOS | AGCCCTTTACTTGACCTCCT | TCCATCTTTCACCCACTTGC | 58°C | 100% |
| MCP-1 | GGCTGAGACTAACCCAGAAAC | GAATGAAGGTGGCTGCTATGA | 58°C | 95% |
| IFN-γ | GGGTTCTCTTGGCTGTTACT | GAGTTCCATTATCCGCTACATCT | 58°C | 92% |
| IL-8 | GAGACAGCAGAGCACACAAG | ACACACAGTGAGATGGTTCC | 58°C | 100% |
