# Supplementary material for: Breadfruit flour is a healthy option for modern foods and food security
Source: PLoS One. 2020 Jul 23;15(7):e0236300. doi: 10.1371/journal.pone.0236300 (PMC7377419; doi:10.1371/journal.pone.0236300)
Supplement: S3 Table — (DOCX) [file pone.0236300.s003.docx]

**S 3 Table. Comparison of nutritional value between the breadfruit (BF) diet and 5LG4 diet.**

| **Title** | **Unit** | **5LG4 diet** | **BF diet** |
| --- | --- | --- | --- |
| Calories USA | Cal/100g | 363 | 357 |
| Calories Canada | Cal/100g | 328 | 330 |
| Total fat as triglycerides by GC | g/100g | 6.53 | 5.93 |
| Saturated fatty acid | g/100g | 1.41 | 1.42 |
| cis-Monounsaturated fatty acid | g/100g | 1.49 | 1.45 |
| cis-Polyunsaturated fatty acid | g/100g | 3.26 | 2.71 |
| Omega-6 fatty acids | g/100g | 2.85 | 2.3 |
| Omega-3 fatty acids | g/100g | 0.41 | 0.41 |
| Trans fatty acids | g/100g | 0.03 | 0.05 |
| Conjugated linoleic acid | g/100g | <0.01 | <0.01 |
| Cholesterol | mg/100g | 28.6 | 23.6 |
| Sodium | mg/100g | 291 | 223 |
| Carbohydrates | g/100g | 56.7 | 60.1 |
| Total dietary fibre | g/100g | 17.6 | 13.4 |
| Total sugars | g/100g | 1.9 | 4.3 |
| Fructose | g/100g | 0.24 | 1 |
| Glucose | g/100g | <0.2 | 0.79 |
| Sucrose | g/100g | 1.6 | 2.5 |
| Maltose | g/100g | <0.5 | <0.5 |
| Lactose | g/100g | <0.5 | <0.5 |
| Protein | g/100g | 19.39 | 15.8 |
| Protein factor |  | 6.25 | 6.25 |
| Total vitamin A | RE/100g | 71 | <20 |
| Retinol | IU/100g | 169 | <50 |
| Beta carotene | IU/100g | 200 | <50 |
| Calcium | mg/100g | 1190 | 1260 |
| Iron | mg/100g | 39.4 | 40.4 |
| Ash | g/100g | 6.94 | 7.18 |
| Moisture | g/100g | 10.43 | 10.95 |
| Vitamin C | mg/g | <1.0 | <1.0 |
| Vanadium | ppm (w/w) | 1.8 | 2.65 |
| Zinc | ppm (w/w) | 65.7 | 54 |
| Zirconium | ppm (w/w) | 0.5 | 0.8 |
| Iodine | ppm (w/w) | 2.13 | 1.56 |
| Niacin | mg/100g | 11.5 | 6.9 |
| Vitamin B1.HCl | mg/100g | 3.2 | 1.6 |
| Vitamin B12 | mcg/100g | 5.5 |  |
| Vitamin B2 | mg/100g | 0.91 | 0.78 |
| Vitamin B6 | mg/100g | 0.79 | 0.67 |
| Vitamin E | IU/100g | 3 | 3.3 |
|  | mg/100g | 2 | 2.2 |
| Biotin | ppm (w/w) | 35.6 | 32.8 |
| Boron | ppm (w/w) | 2.6 | 5.9 |
| Aluminum | ppm (w/w) | 147 | 203 |
| Antimony | ppm (w/w) | 0.02 | 0.04 |
| Arsenic | ppm (w/w) | 0.7 | 0.68 |
| Barium | ppm (w/w) | 7.71 | 6.4 |
| Beryllium | ppm (w/w) | 0.04 | 0.07 |
| Bismuth | ppm (w/w) | <0.02 | <0.02 |
| Cadmium | ppm (w/w) | 0.076 | 0.098 |
| Calcium | ppm (w/w) | 13400 | 17200 |
| Chromium | ppm (w/w) | 1.54 | 2.23 |
| Cobalt | ppm (w/w) | 0.56 | 0.66 |
| Copper | ppm (w/w) | 7.49 | 7.88 |
| Iron | ppm (w/w) | 345 | 486 |
| Lead | ppm (w/w) | 0.11 | 0.24 |
| Lithium | ppm (w/w) | 0.2 | 0.3 |
| Magnesium | ppm (w/w) | 2190 | 1650 |
| Manganese | ppm (w/w) | 120 | 143 |
| Mercury | ppm (w/w) | 0.005 | 0.007 |
| Molybdenum | ppm (w/w) | 1.57 | 0.94 |
| Nickel | ppm (w/w) | 1.01 | 1.41 |
| Phosphorus | ppm (w/w) | 9390 | 10400 |
| Potassium | ppm (w/w) | 7160 | 11000 |
| Selenium | ppm (w/w) | 0.4 | 0.5 |
| Silver | ppm (w/w) | <0.02 | <0.02 |
| Sodium | ppm (w/w) | 2810 | 2060 |
| Strontium | ppm (w/w) | 12.9 | 16.8 |
| Thallium | ppm (w/w) | <0.01 | 0.01 |
| Thorium | ppm (w/w) | 0.2 | 0.2 |
| Tin | ppm (w/w) | 0.02 | 0.02 |
| Titanium | ppm (w/w) | 19.2 | 24.6 |
| Uranium | ppm (w/w) | 0.37 | 0.67 |
