# Supplementary material for: Breadfruit flour is a healthy option for modern foods and food security
Source: PLoS One. 2020 Jul 23;15(7):e0236300. doi: 10.1371/journal.pone.0236300 (PMC7377419; doi:10.1371/journal.pone.0236300)
Supplement: S4 Table — (DOCX) [file pone.0236300.s004.docx]

**S 4 Table. Comparison of average body composition and tissue weight between the BF-fed (breadfruit-fed) mice and 5LG4-fed mice.**

| Sex | 5LG4 diet | | BF diet | | Male mice | | | | Female mice | | | |
| --- | --- | --- | --- | --- | --- | --- | --- | --- | --- | --- | --- | --- |
| Diet |  |  |  |  | 5LG4 diet | | BF diet | | 5LG4 Diet | | BF Diet | |
| Name | Mean | SE | Mean | SE | Mean | SE | Mean | SE | Mean | SE | Mean | SE |
| Pancreas (g) | 0.11 | 0.007 | 0.11 | 0.006 | 0.12 | 0.011 | 0.13 | 0.006 | 0.10 | 0.010 | 0.10 | 0.008 |
| Duodenum (g) | 0.28 | 0.024 | 0.22 | 0.021 | 0.28 | 0.037 | 0.22 | 0.025 | 0.20a | 0.027 | 0.31b | 0.028 |
| Jejunum (g) | 0.25 | 0.042 | 0.21 | 0.012 | 0.23 | 0.024 | 0.22 | 0.018 | 0.27 | 0.084 | 0.19 | 0.013 |
| Ileum (g) | 0.20 | 0.037 | 0.18 | 0.008 | 0.22 | 0.071 | 0.19 | 0.011 | 0.18 | 0.028 | 0.17 | 0.010 |
| Colon (g) | 0.15 | 0.009 | 0.16 | 0.012 | 0.16 | 0.012 | 0.16 | 0.018 | 0.13 | 0.013 | 0.15 | 0.017 |
| Testes (g) | 0.18 | 0.024 | 0.18 | 0.024 | 0.18 | 0.004 | 0.18 | 0.005 | / | / | / | / |
| Epididymis (g) | 0.05 | 0.007 | 0.05 | 0.007 | 0.05 | 0.007 | 0.05 | 0.005 | / | / | / | / |
| Uterus (g) | 0.09 | 0.014 | 0.08 | 0.011 | / | / | / | / | 0.09 | 0.016 | 0.08 | 0.010 |
| Mammary gland (g) | 0.22 | 0.012 | 0.20 | 0.015 | 0.22 | 0.015 | 0.18 | 0.016 | 0.23 | 0.021 | 0.23 | 0.023 |
| Pituitary gland (g) | 0.002 | 0.001 | 0.002 | 0.000 | 0.002 | 0.001 | 0.002 | 0.001 | 0.002 | 0.001 | 0.001 | 0.000 |
| Adrenal gland (g) | 0.01 | 0.001 | 0.01 | 0.001 | 0.01 | 0.001 | 0.01 | 0.001 | 0.01 | 0.001 | 0.01 | 0.000 |
| Ears (g) | 0.04 | 0.005 | 0.04 | 0.004 | 0.06 | 0.006 | 0.04 | 0.007 | 0.03 | 0.004 | 0.04 | 0.005 |
| Tail tip (g) | 0.06 | 0.006 | 0.13 | 0.047 | 0.06 | 0.007 | 0.12 | 0.058 | 0.06 | 0.010 | 0.13 | 0.078 |
| Femur (g) | 0.06 | 0.003 | 0.09 | **0.009** | **0.07a** | **0.005** | **0.08b** | 0.002 | 0.06 | 0.003 | 0.09 | 0.019 |
| Thymus (g) | 0.03 | 0.004 | 0.03 | 0.005 | 0.03 | 0.006 | 0.03 | 0.007 | 0.03 | 0.006 | 0.04 | 0.005 |
| Spleen (g) | 0.06 | 0.002 | 0.06 | 0.003 | 0.06 | 0.002 | 0.07 | 0.003 | 0.06 | 0.004 | 0.06 | 0.005 |
| Lymph node (g) | 0.01 | 0.003 | 0.01 | 0.002 | 0.02 | 0.005 | 0.01 | 0.003 | 0.01 | 0.004 | 0.01 | 0.002 |
| Salivary gland (g) | 0.12 | 0.011 | 0.12 | 0.013 | 0.14 | 0.017 | 0.14 | 0.016 | 0.11 | 0.015 | 0.10 | 0.019 |
| Bladder (g) | 0.02 | 0.002 | 0.02 | 0.002 | 0.02 | 0.001 | 0.03 | 0.003 | 0.02 | 0.003 | 0.01 | 0.002 |

Numbers followed by different letter in each section are significant difference at alpha=0.05, using 2 sample t test.
