# Supplementary material for: Breadfruit flour is a healthy option for modern foods and food security
Source: PLoS One. 2020 Jul 23;15(7):e0236300. doi: 10.1371/journal.pone.0236300 (PMC7377419; doi:10.1371/journal.pone.0236300)
Supplement: S5 Table — (DOCX) [file pone.0236300.s005.docx]

**S 5 Table. Histological examination of ileum morphology.**

| Sex | 5LG4 diet | | BF diet | | Male mice | | | | Female mice | | | |
| --- | --- | --- | --- | --- | --- | --- | --- | --- | --- | --- | --- | --- |
| Diet |  |  |  |  | 5LG4 diet | | BF diet | | 5LG4 diet | | BF diet | |
| Title | Mean | SE | Mean | SE | Mean | SE | Mean | SE | Mean | SE | Mean | SE |
| Distance between villi | 6.43 | 0.95 | 6.28 | 0.72 | 6.26 | 0.94 | 5.92 | 0.49 | 6.60 | 1.50 | 6.63 | 1.23 |
| Height of villus (µm) | 110.2 | 9.23 | 117.3 | 6.66 | 120.4 | 10.41 | 125.5 | 6.46 | 99.98 | 11.74 | 109.0 | 8.90 |
| Thickness of villus (µm) | 51.04 | 2.83 | 52.41 | 2.50 | 53.36 | 1.49 | 55.59 | 3.51 | 48.73 | 4.81 | 49.23 | 2.14 |
| Crypt depth (µm) | 68.75 | 5.00 | 56.66 | 2.31 | 70.38 | 7.93 | 61.92 | 2.07 | 67.12 | 4.85 | 51.40 | 0.78 |
| Thickness of lamina propria (µm) | 20.71 | 1.98 | 18.01 | 1.93 | 21.07 | 1.30 | 19.05 | 2.91 | 20.36 | 3.46 | 16.97 | 2.00 |
| Epithelium thickness (µm) | 15.48 | 1.09 | 17.26 | 0.60 | 16.45 | 1.63 | 18.40 | 0.52 | 14.50 | 1.02 | 16.13 | 0.60 |
| Length of mucosa (µm) | 171.3 | 13.05 | 170.2 | 8.00 | 183.3 | 16.03 | 182.8 | 7.33 | 159.3 | 16.33 | 157.7 | 9.57 |
| Length of submucosa (µm) | 9.90 | 0.68 | 9.26 | 0.52 | 8.86 | 0.41 | 9.44 | 0.60 | 10.95 | 0.95 | 9.08 | 0.76 |
| Length of muscularis externa (µm) | 21.24 | 2.07 | 21.25 | 1.50 | 22.08 | 3.40 | 23.50 | 2.28 | 20.39 | 1.77 | 19.01 | 0.40 |
| Number of goblet cells | 6 | 0 | 6 | 0 | 7 | 0 | 7 | 0 | 5 | 0 | 6 | 0 |
| Number of red blood cells | 5 | 2 | 5 | 2 | 5 | 1 | 3 | 2 | 6 | 3 | 7 | 2 |

Numbers followed by different letter in each section are significant difference at alpha=0.05, using 2 sample t test.
