# Supplementary figures and images for: Breadfruit flour is a healthy option for modern foods and food security
Source: PLoS One. 2020 Jul 23;15(7):e0236300. doi: 10.1371/journal.pone.0236300 (PMC7377419; doi:10.1371/journal.pone.0236300)

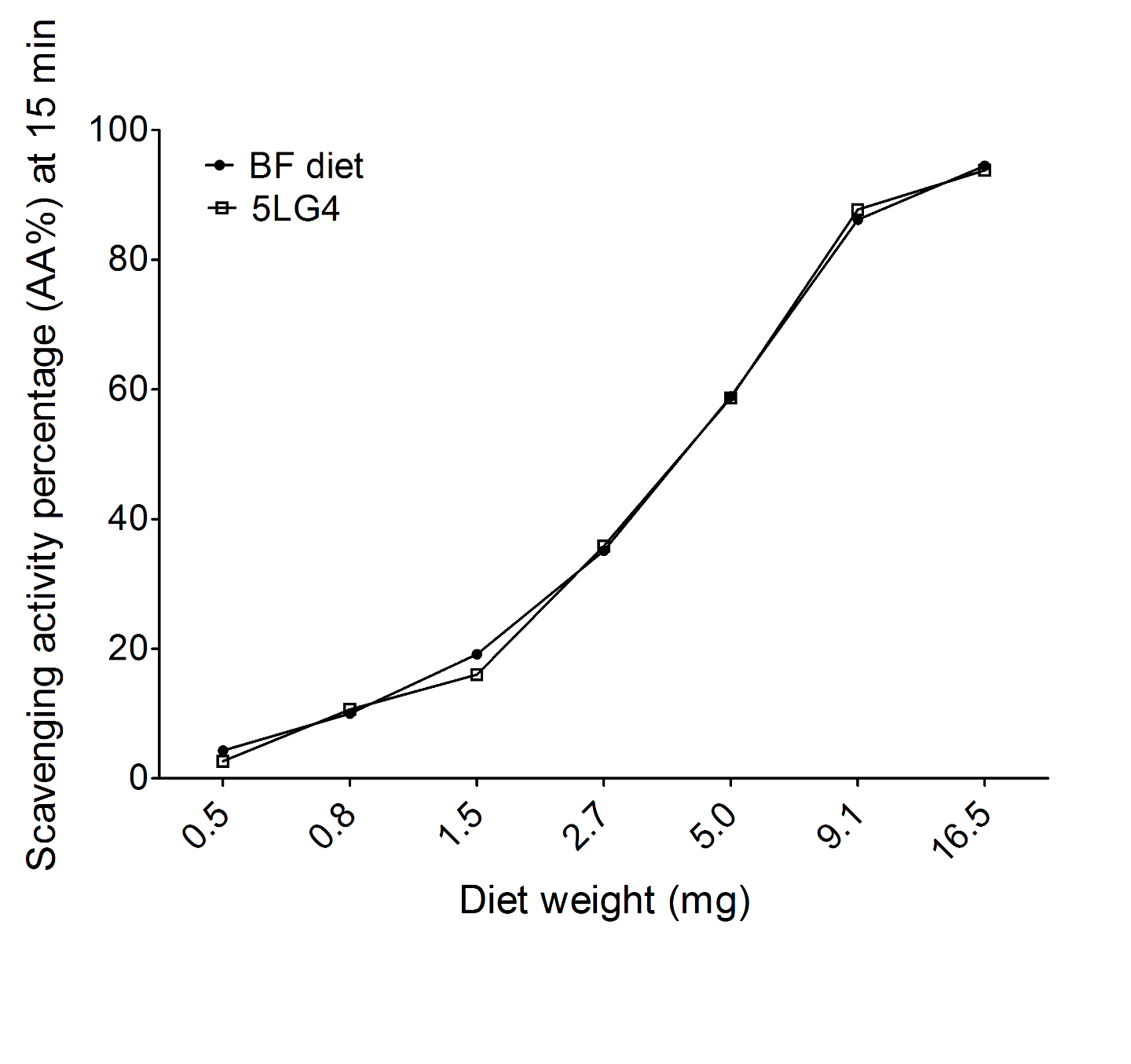


S 1 Fig

Supplement: S1 Fig — Bars represents standard error calculated from the three replicates. (DOCX) [file pone.0236300.s006.docx]

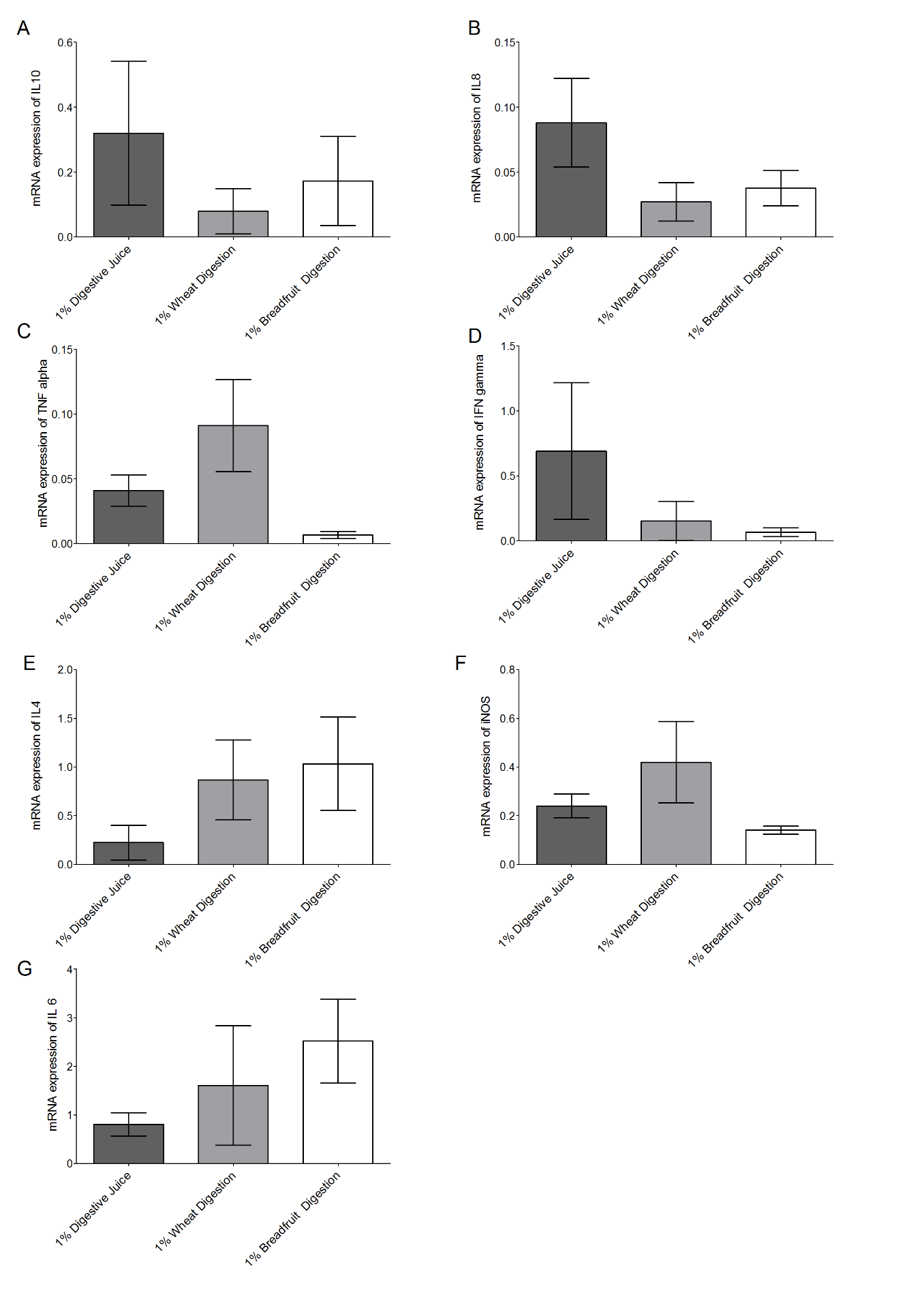


S 2 Fig

Supplement: S2 Fig — (A) IL-10. (B). IL 8. (C) TNF-α. (D) IFN-γ. (E). IL-4. (F) iNOS. (G) IL-6. (DOCX) [file pone.0236300.s007.docx]

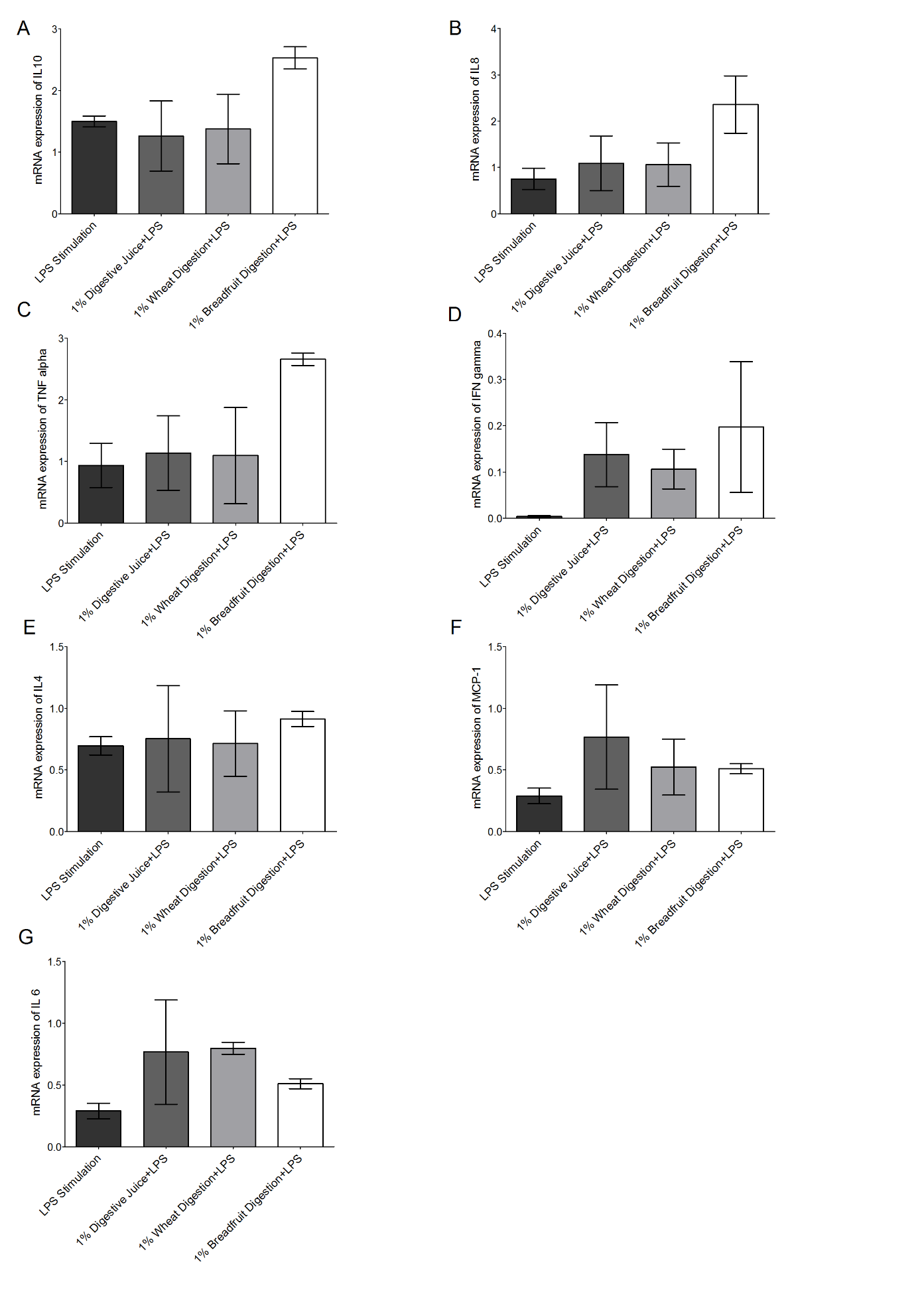


S 3 Fig

Supplement: S3 Fig — (A) IL-10. (B). IL 8. (C) TNF-α. (D) IFN-γ. (E). IL-4. (F) MCP-1. (G) IL-6. (DOCX) [file pone.0236300.s008.docx]

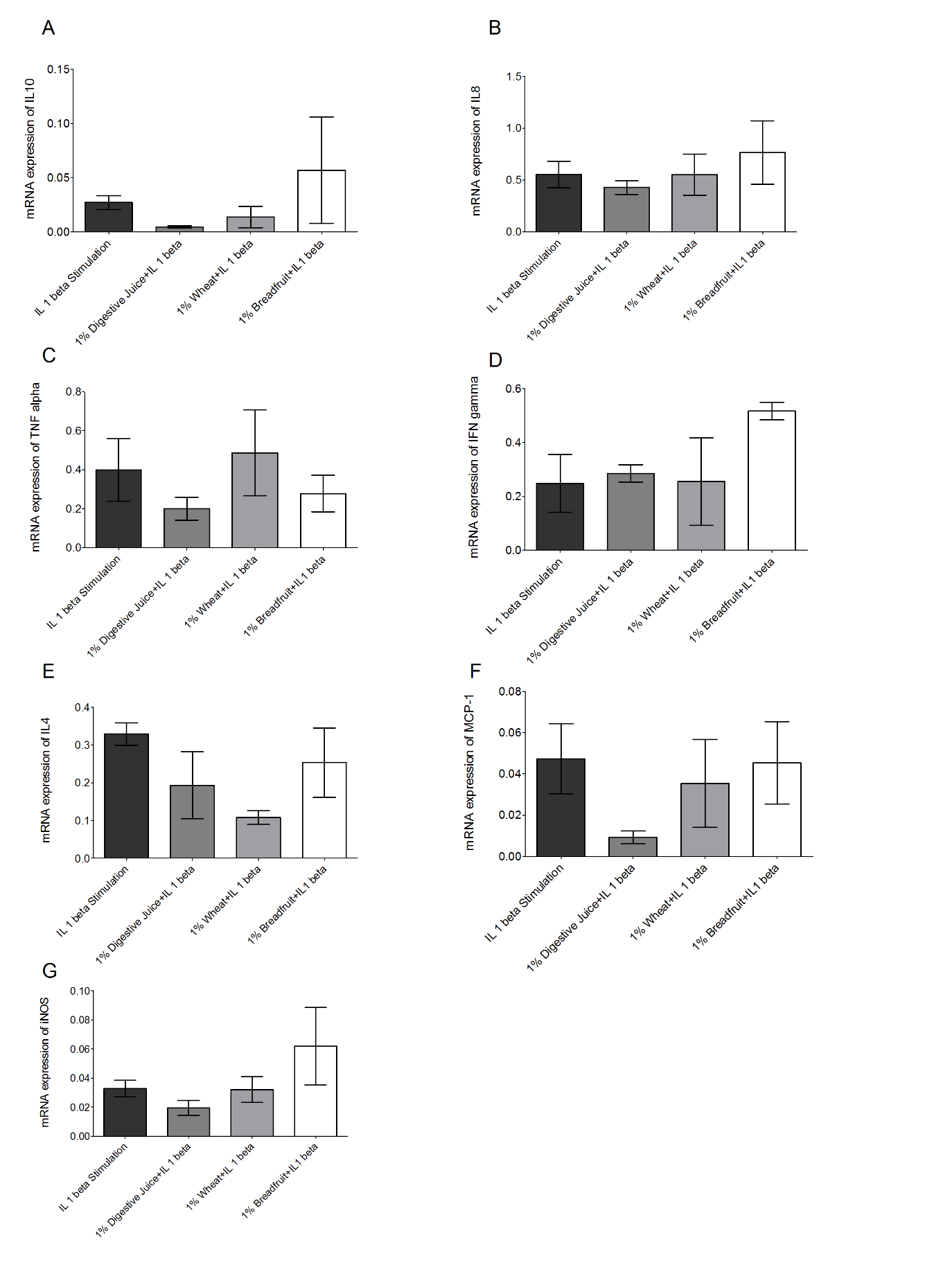


S 4 Fig

Supplement: S4 Fig — (A) IL-10. (B). IL 8. (C) TNF-α. (D) IFN-γ. (E). IL-4. (F) MCP-1. (G) iNOS. (DOCX) [file pone.0236300.s009.docx]

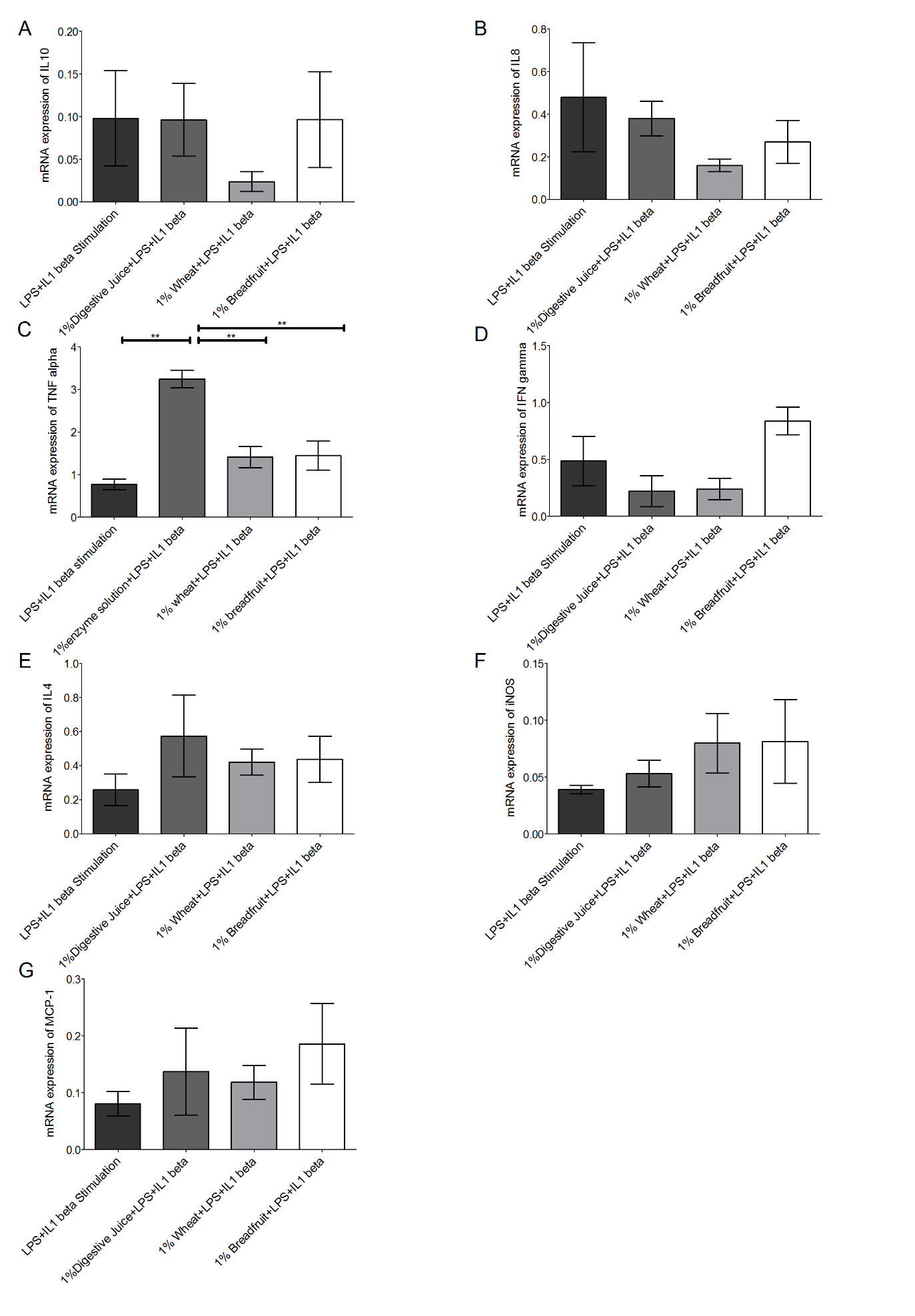


S 5 Fig

Supplement: S5 Fig — (A) IL-10. (B). IL 8. (C) TNF-α. (D) IFN-γ. (E). IL-4. (F) iNOS. (G) MCP-1. (DOCX) [file pone.0236300.s010.docx]

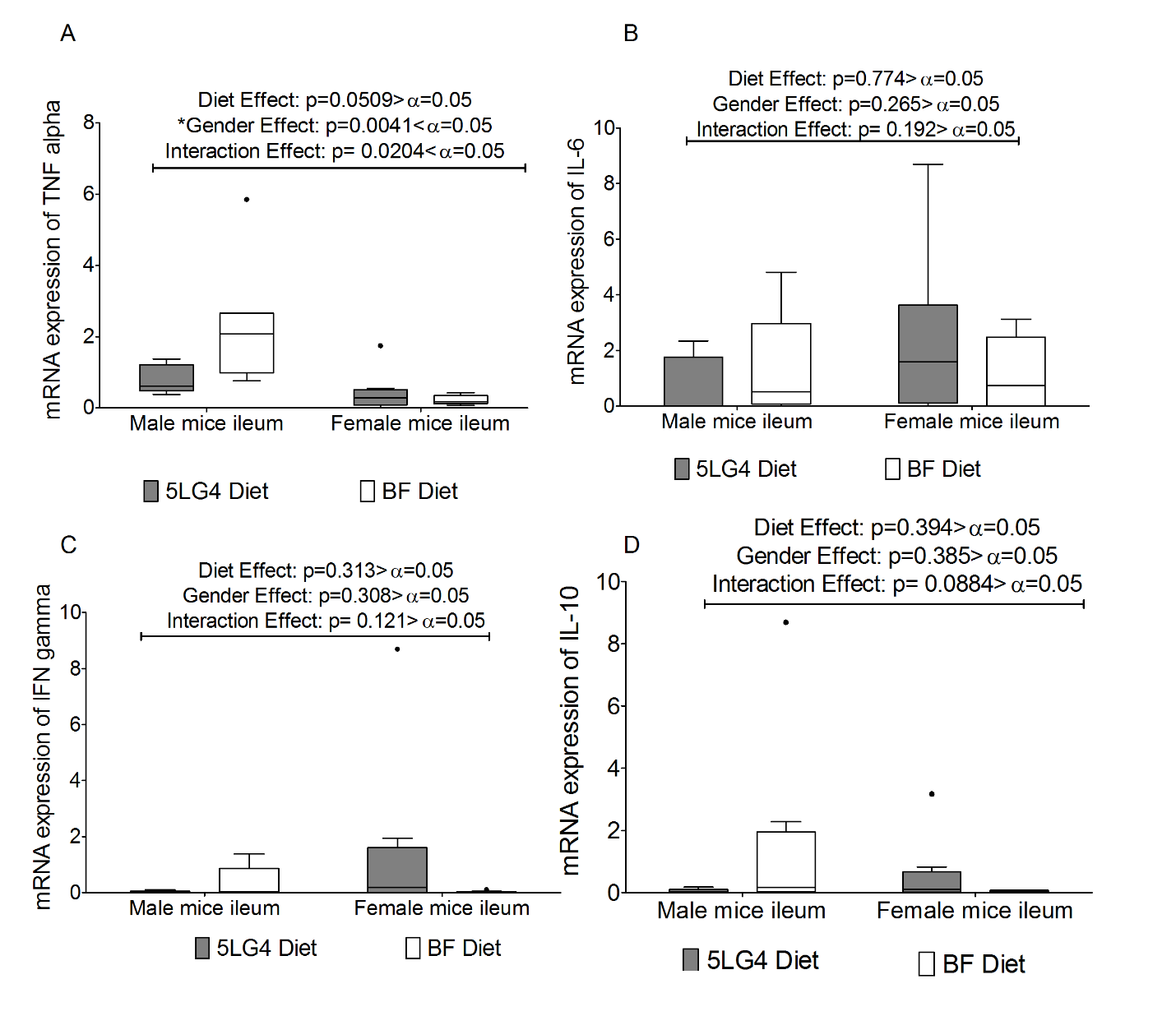


S 6 Fig

Supplement: S6 Fig — (A) TNF-α. (B) IL-6. (C) IFN-γ. (D) IL-10. (DOCX) [file pone.0236300.s011.docx]

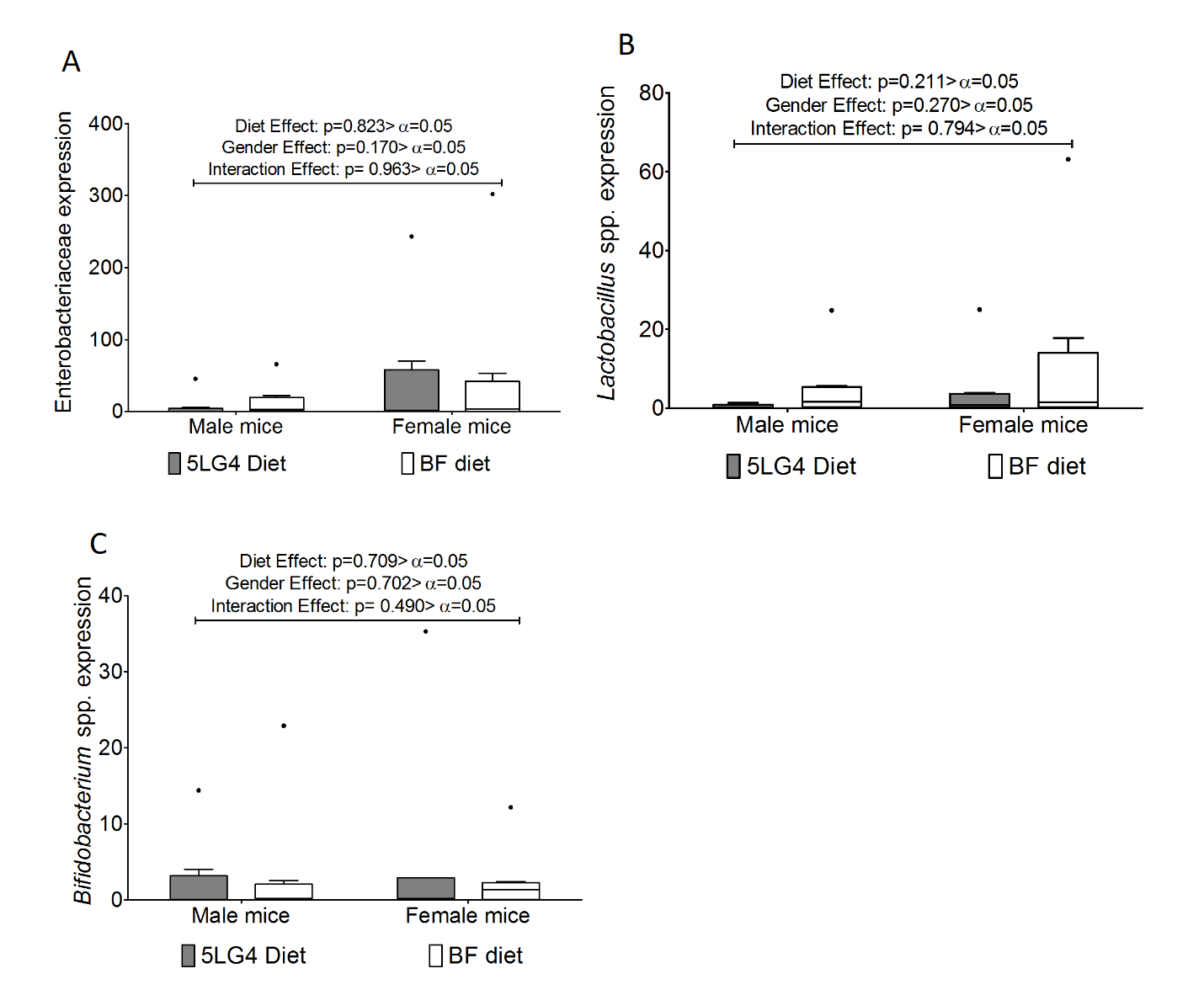


S 7 Fig

Supplement: S7 Fig — (A) Enterobacteriacae. (B) Lactobacillus spp. (C) Bifidobacterium spp. (DOCX) [file pone.0236300.s012.docx]
